# Genome-Wide Meta-Analysis of Sciatica in Finnish Population

**DOI:** 10.1371/journal.pone.0163877

**Published:** 2016-10-20

**Authors:** Susanna Lemmelä, Svetlana Solovieva, Rahman Shiri, Christian Benner, Markku Heliövaara, Johannes Kettunen, Verneri Anttila, Samuli Ripatti, Markus Perola, Ilkka Seppälä, Markus Juonala, Mika Kähönen, Veikko Salomaa, Jorma Viikari, Olli T. Raitakari, Terho Lehtimäki, Aarno Palotie, Eira Viikari-Juntura, Kirsti Husgafvel-Pursiainen

**Affiliations:** 1 Health and Work Ability, Finnish Institute of Occupational Health, 00250 Helsinki, Finland; 2 Institute for Molecular Medicine Finland (FIMM), 00014 University of Helsinki, Helsinki, Finland; 3 Department of Public Health, 00014 University of Helsinki, Helsinki, Finland; 4 Population Health Unit, National Institute for Health and Welfare, 00251 Helsinki, Finland; 5 Faculty of Medicine, Institute of Health Sciences, University of Oulu, 90220 Oulu, Finland; 6 NMR Metabolomics Laboratory, University of Eastern Finland, Kuopio, Finland; 7 National Institute for Health and Welfare, Helsinki, Finland; 8 Analytic and Translational Genetics Unit, Department of Medicine, Massachusetts General Hospital, Boston, Massachusetts 02114, United States of America; 9 Program in Medical and Population Genetics, Broad Institute of MIT and Harvard, Cambridge, Massachusetts 02142, United States of America; 10 Wellcome Trust Sanger Institute, Wellcome Trust Genome Campus, Cambridge, CB10 1SA, United Kingdom; 11 Public Health Genomics Unit, Department of Chronic Disease Prevention, National Institute for Health and Welfare, 00271 Helsinki, Finland; 12 The Estonian Genome Center, University of Tartu, 51010 Tartu, Estonia; 13 Department of Clinical Chemistry, Fimlab Laboratories, University of Tampere School of Medicine, 33520 Tampere, Finland; 14 Division of Medicine, Turku University Hospital, 20521 Turku, Finland; 15 Department of Medicine, University of Turku, 20521 Turku, Finland; 16 Department of Clinical Physiology, Tampere University Hospital, 33521 Tampere, Finland; 17 Department of Health, National Institute for Health and Welfare, 00251 Helsinki, Finland; 18 Research Centre of Applied and Preventive Cardiovascular Medicine, University of Turku, 20520 Turku, Finland; 19 Department of Clinical Physiology and Nuclear Medicine, Turku University Hospital, 20521 Turku, Finland; 20 Psychiatric & Neurodevelopmental Genetics Unit, Department of Psychiatry, Massachusetts General Hospital, Boston, Massachusetts 02114, United States of America; 21 Disability Prevention Centre, Finnish Institute of Occupational Health, 00250 Helsinki, Finland; Wake Forest School of Medicine, UNITED STATES

## Abstract

Sciatica or the sciatic syndrome is a common and often disabling low back disorder in the working-age population. It has a relatively high heritability but poorly understood molecular mechanisms. The Finnish population is a genetic isolate where small founder population and bottleneck events have led to enrichment of certain rare and low frequency variants. We performed here the first genome-wide association (GWAS) and meta-analysis of sciatica. The meta-analysis was conducted across two GWAS covering 291 Finnish sciatica cases and 3671 controls genotyped and imputed at 7.7 million autosomal variants. The most promising loci (p<1x10^-6^) were replicated in 776 Finnish sciatica patients and 18,489 controls. We identified five intragenic variants, with relatively low frequencies, at two novel loci associated with sciatica at genome-wide significance. These included chr9:14344410:I (rs71321981) at 9p22.3 (*NFIB* gene; p = 1.30x10^-8^, MAF = 0.08) and four variants at 15q21.2: rs145901849, rs80035109, rs190200374 and rs117458827 (*MYO5A*; p = 1.34x10^-8^, MAF = 0.06; p = 2.32x10^-8^, MAF = 0.07; p = 3.85x10^-8^, MAF = 0.06; p = 4.78x10^-8^, MAF = 0.07, respectively). The most significant association in the meta-analysis, a single base insertion rs71321981 within the regulatory region of the transcription factor *NFIB*, replicated in an independent Finnish population sample (p = 0.04). Despite identifying 15q21.2 as a promising locus, we were not able to replicate it. It was differentiated; the lead variants within 15q21.2 were more frequent in Finland (6–7%) than in other European populations (1–2%). Imputation accuracies of the three significantly associated variants (chr9:14344410:I, rs190200374, and rs80035109) were validated by genotyping. In summary, our results suggest a novel locus, 9p22.3 (*NFIB*), which may be involved in susceptibility to sciatica. In addition, another locus, 15q21.2, emerged as a promising one, but failed to replicate.

## Introduction

Low back pain is a global health problem affecting all age groups [1, 2]. Sciatica–usually a clinical manifestation of lumbar disc herniation (OMIM 603932)—is a common low back disorder with a population prevalence of about 5%; it is often disabling in the working age population [3, 4]. Sciatica is a complex disorder, with both genetic and environmental factors involved [4, 5]. Sciatic pain or lumbar radicular pain–the typical symptom of sciatica–is defined as pain radiating from the back down to the leg, usually caused by compression or irritation of one of the lumbosacral nerve roots [6–9].

Twin and family studies have revealed a substantial genetic component in low back disorders with heritability estimates of approximately 20–40% for sciatica and 35–75% for lumbar disc degeneration (OMIM 603932) [10–12]. Candidate gene studies of low back disorders have typically focused on functional genes that associate with cartilage structure and stability, pain signaling, obesity, or inflammation [13–15].

The Finnish population is one of the most thoroughly characterized genetic isolate. It has its ancestry in a small founder population, followed by several bottle neck events and genetic drift that has led to the enrichment of certain rare and low frequency variants that are almost absent in many other European populations [16]. Presently, there are several representative Finnish population cohorts with genome-wide data available, and these have contributed to an array of successful large GWAS consortia [17–25].

The current study was conducted in two of the Finnish population cohorts, the Young Finns Study (YFS) and Health 2000 Study (H2000), both with data on physician-diagnosed sciatica available. We rationalized that the unique Finnish population features together with the representative population-based studies allow us to investigate possible influence of not only common variants but also discover rare or low frequency variants involved in the development of sciatica.

In the present study, we performed the first genome-wide association studies (GWAS) and GWAS meta-analysis of sciatica (291 sciatica cases and 3671 controls), utilizing the special benefits of the Finnish population isolate and the power of 1000 Genomes imputation. We validated imputation accuracies of three significantly associated variants by genotyping them in the discovery cohorts. The most promising loci (p < 1x10^-6^) were replicated in an independent population-based sample of 776 Finnish sciatica patients and 18,489 controls.

## Results

### Genome-wide meta-analysis

**Fig 1:**
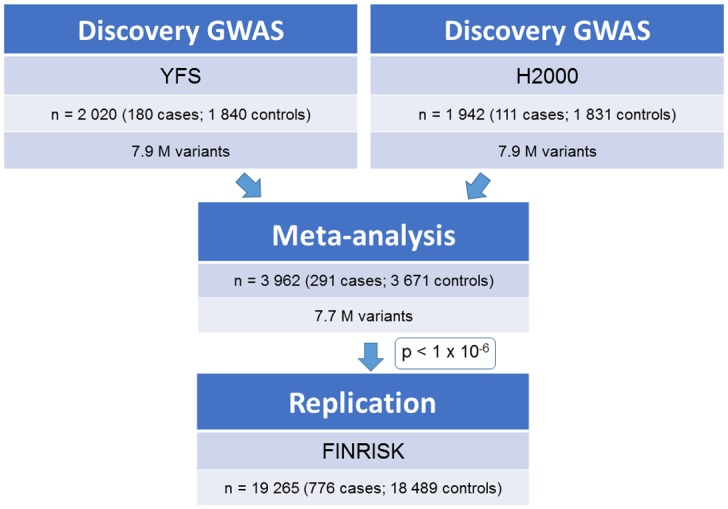
Study design. Two discovery GWAS were conducted in Finnish population-based cohorts, the Young Finns Study (YFS) and the Health 2000 Study (H2000). Meta-analysis was carried out across the discovery GWAS. The most promising variants in meta-analysis (p<1x10^-6^) were replicated in a subsample of the FINRISK Study.

We conducted a meta-analysis of sciatica across two Finnish GWAS covering 291 sciatica cases and 3,671 controls genotyped or imputed at 7.7 million autosomal variants (Fig 1; S1 Table). Both GWAS (YFS and H2000) (see URLs; S1 Text) are based on Finnish population-based cohorts with dense genome-wide genotyping and 1000 Genomes imputation data (Fig 1; Table 1; S1 Table).

**Table 1:** Sample demographics.

Study	Status	N	Age		Female %	BMI		Smoking % (Smokers/Non-smokers)	PA % (Very low or no/Active)
			Mean	S.D.		Mean	S.D.		
YFS	All	2020	37.7	5.0	55	26.0	4.7	23 (450/1468)	23 (455/1561)
	Case	180	39.2	4.8	64	26.6	5.1	28 (48/123)	21 (38/141)
	Control	1840	37.6	5.0	54	25.9	4.7	23 (402/1345)	23 (417/1420)
H2000	All	1942	50.4	10.9	51	27.2	4.5	29 (571/1365)	25 (470/1443)
	Case	111	54.4	10.6	55	28.0	4.7	29 (32/79)	27 (30/80)
	Control	1831	50.1	10.9	50	27.2	4.5	30 (539/1286)	24 (440/1363)
FINRISK	All	19 265	48.1	13.3	55	26.8	4.7	27 (5144/13961)	NA
	Case	776	50.7	12.3	48	27.7	4.5	28 (212/558)	NA
	Control	18 489	48.0	13.3	55	26.8	4.7	27 (4932/13403)	NA

N, Number of individuals; BMI, Body Mass Index; S.D., Standard Deviation; Smoking, Percentage of smokers (Numbers of smokers *vs* non-smokers given for each group); PA, Percentage of subjects with very low or no physical activity (Numbers of those with no physical activity or up to 3 times a month *vs* once a week or more frequently); NA, Not available. YFS, The Cardiovascular Risk in Young Finns Study; H2000, The Health 2000 Study; FINRISK, a subsample (years 1992, 1997, 2002, 2007) of the FINRISK Study. Values given represent those at the time of the questionnaire.

**Fig 2:**
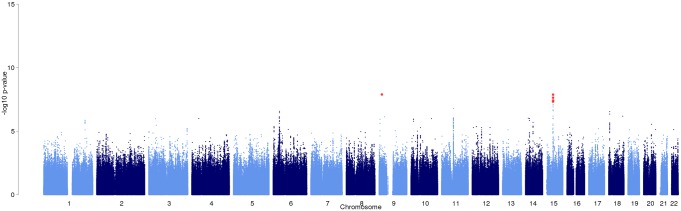
Manhattan plot for meta-analysis of adjusted genome-wide association results. Variants with p-values below the genome-wide significance level (p < 5x10^-8^) are shown in red.

**Fig 3:**
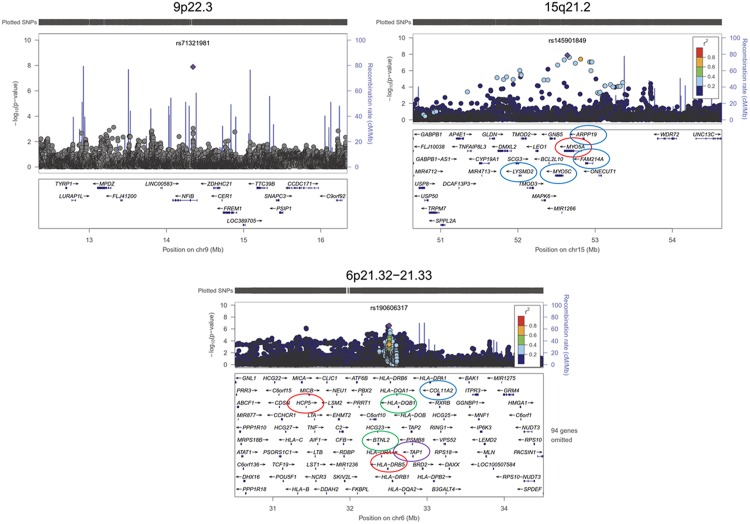
Regional association plots for associated loci in the GWAS meta-analysis of sciatica. The associations along with recombination rates and genes on the region are shown in 2 Mb windows surrounding the lead SNP, to provide a graphical view of the associated region. SNPs are plotted by position on chromosome (x-axis) against association with sciatica (-log_10_ –p-value, y-axis). The lead SNP is shown with a purple diamond. Color intensity of each dot depicting a SNP reflects the extent of LD with the lead SNP, colored red (r^2^<0.8) through blue (r^2^<0.2). The LD has been estimated using 1000 Genomes, Mar2012 release, European population (see URLs). Physical positions are based on of the human genome build 37 (NCBI). **9p22.3:** (*NFIB*) represented by rs71321981 (chr9:14344410:I, p = 1.30x10^-8^). No usable LD information was available for this SNP. **15q21.2:** (*MYO5A*) represented by rs145901849 (p = 1.34x10^-8^). The associated region harbor SNPs in the *MYO5A* (p < 5x10^-8^) (red circle) and SNPs in the surrounding genes *MYO5C*, *LYSMD2*, *ARPP19*, and *FAM214A* (p<1.0x10^-6^) (blue circles). **6p21.32:** (*HLA-DRB5*) represented by rs115488695 (p = 3.58x10^-7^). The *HLA* gene region (6p21.32) has previously been associated with musculoskeletal disorders; SNPs (rs2187689, rs7767277) nearby *TAP1* (violet circle) were associated with lumbar disc degeneration in the meta-analysis of Northern European individuals [26], two SNPs (rs7775228, rs10947262) within *BTNL2* and nearby *HLA-DQB1* genes (green circles) were associated with knee osteoarthritis in a Japanese GWAS [27), and two SNPs (rs2076311 and rs1799907) within *COL11A2* (blue circle) were associated with magnetic resonance-determined disc signal intensity [28], and with degenerative lumbar spinal stenosis with radicular pain in Finnish individuals [29].

In the meta-analysis of GWAS data, a total of five novel variants within two loci achieved genome-wide significance (p<5x10^-8^). These were insertion chr9:14344410:I (also known as rs71321981) at 9p22.3 (p = 1.30x10^-8^; MAF 0.08) and rs145901849, rs80035109, rs190200374 and rs117458827 at 15q21.2 (p = 1.34x10^-8^, MAF = 0.06; 2.32x10^-8^, MAF = 0.07; 3.85x10^-8^, MAF = 0.06; 4.78x10^-8^, MAF = 0.07, respectively). The between study heterogeneity (*I*^*2*^) ranged between 0–0.63 and the imputation quality was high (0.77–0.99) for the associated variants (Table 1; S2 and S3 Tables). From these, the insertion chr9:14344410:I (rs71321981) is a regulation region variant in the first intron of the *NFIB* gene. The 15q21.2 variants (an intronic regulation region variant rs145901849; intronic SNPs rs80035109 and rs190200374, as well as a 3’ UTR SNP rs117458827) are located within ~200kb region in the *MYO5A* gene and are in a strong linkage disequilibrium (LD) (r^2^≥0.81) (Table 1; S4 Table; Figs 2 and 3; 1000 Genomes Project; see URLs).

All five significantly associated variants have relatively low frequencies (MAF≤0.08). The insertion chr9:14344410:I at 9p22.3 (*NFIB*) has similar frequencies in the Finnish and other European populations (7% and 8%, respectively), whereas all four lead variants at the locus 15q21.2 *(MYO5A*) were more frequent in Finnish population (6–7%) than in other Europeans (0–2%, respectively) (Table 2; S5 Table; 1000 Genomes Project; see URLs).

**Table 2:** SNPs exhibiting genome-wide significant association with sciatica in the GWAS meta-analysis.

SNP	Type	Chr	Position^&^ (bp)	Gene	EA/OA	Analysis (GWAS/Replication)	EAF			Imput. quality^#^	OR* (95% CI)	beta	SE	P value*	P_het_	*I*^*2*^
							all	case	cntrl							
chr9:14344410:I; rs71321981	regulatory region	9p22.3	14344410	*NFIB*	AG/A	YFS	0.08	0.13	0.07	0.78	3.17 (1.94–5.18)	1.15	0.25	4.09x10^-6^		
						H2000	0.07	0.13	0.07	0.77	2.89 (1.62–5.19)	1.06	0.30	3.54x10^-4^		
						Meta-analysis	0.08	-	-	-	3.05 (2.08–4.49)	1.12	0.20	1.30x10^-8^	0.82	0
						Replication^@^	0.07	0.08	0.07	0.59	1.17 (0.97–1.40)	0.16	0.09	0.04		
rs145901849	regulatory region	15q21.2	52640539	*MYO5A*	T/C	YFS	0.06	0.12	0.05	0.91	3.84 (2.38–6.20)	1.35	0.24	3.83x10^-8^		
						H2000	0.06	0.09	0.06	0.93	2.01 (1.08–3.75)	0.70	0.32	2.8x10^-2^		
						Meta-analysis	0.06	-	-	-	3.04 (2.07–4.45)	1.11	0.19	1.34x10^-8^	0.11	0.61
						Replication^@^	0.003	0.004	0.003	0.53	1.42 (0.66–3.04)	0.35	0.39	0.22		
rs80035109	intronic	15q21.2	52665890	*MYO5A*	C/T	YFS	0.07	0.14	0.07	0.97	3.01 (1.97–4.59)	1.10	0.22	3.73x10^-7^		
						H2000	0.07	0.11	0.07	0.97	2.10 (1.19–3.68)	0.74	0.29	9.96x10^-3^		
						Meta-analysis	0.07	-	-	-	2.65 (1.88–3.72)	0.97	0.17	2.32x10^-8^	0.32	0
						Replication^@^	0.07	0.07	0.07	0.83	1.11 (0.91–1.35)	0.10	0.10	0.25		
rs190200374	intronic	15q21.2	52811959	*MYO5A*	T/G	YFS	0.06	0.13	0.06	0.84	3.64 (2.28–5.83)	1.29	0.24	6.72x10^-8^		
						H2000	0.06	0.09	0.06	0.87	1.88 (1.01–3.51)	1.88	0.32	4.7x10^-2^		
						Meta-analysis	0.06	-	-	-	2.89 (1.98–4.21)	1.06	0.19	3.85x10^-8^	0.10	0.63
						Replication^@^	0.07	0.07	0.07	0.93	1.10 (0.90–1.34)	0.10	0.10	0.33		
rs117458827	3’UTR	15q21.2	52600066	*MYO5A*	A/G	YFS	0.07	0.14	0.07	0.99	2.85 (1.88–4.32)	1.05	0.21	8.19x10^-7^		
						H2000	0.07	0.11	0.07	0.99	2.05 (1.19–3.54)	0.72	0.28	9.98x10^-3^		
						Meta-analysis	0.07	-	-	-	2.53 (1.81–3.53)	0.93	0.17	4.78x10^-8^	0.35	0
						Replication^@^	0.07	0.08	0.07	0.92	1.06 (0.88–1.29)	0.06	0.10	0.51		

The respective data from the two discovery GWAS (YFS, H2000), meta-analysis and replication cohort are shown.

^&^Chromosomal positions are based on NCBI build 37;

^#^Imputation quality score from IMPUTE;

*Additive model, adjusted for seven principal components, age and gender;

^@^The FINRISK Study.

Abbreviations: SNP, single nucleotide polymorphism; Type, type of variant; Chr, chromosomal locus; EA, effect allele; OA, other allele; EAF, effect allele frequency;—, not applicable; OR (95% CI), odds ratio (95% confidence interval); beta, effect size; SE, standard error of beta; P_het_, Cochran’s heterogeneity statistic’s p-value; *I*^*2*^, heterogeneity index.

In addition, 176 variants at 30 loci showed suggestive associations with sciatica (p<1.0x10^-5^) (Fig 2; S1 Fig; S2 and S3 Tables). Of these, 45 variants were within a 1.4 Mb region at 6p21.32–33 in the *HLA* gene region, with six lead SNPs having p<1x10^-6^. Four of these (s115949512, rs3094014, rs114615271, rs115688765) were in perfect LD (r^2^≥0.97) within or nearby the *HCP5* gene at 6p21.33, and two (rs190606317 and rs115488695) were in moderate LD (r^2^≥0.31) within or nearby the *HLA-DRB5* gene at 6p21.32 (S6 Table). The locus 6p21.32 has previously been associated with lumbar disc degeneration and osteoarthritis (OMIM 165720) [26, 27] (Fig 3).

The genome-wide inflation factor in the meta-analysis was low (λ_GC_ = 0.99). Manhattan and Quantile-Quantile (QQ) -plots for meta-analysis of adjusted genome-wide association results (adjusted for age, sex and the seven principal components of the genetic data) are shown in Fig 2 and S1 Fig. Regional plots for the associated loci are shown in the Fig 3. Manhattan and QQ -plots of adjusted individual GWAS are shown in S2, S3, S4 and S5 Figs.

### Replication

From the meta-analysis, we selected 30 most promising SNPs (p<1x10^-6^) representing eight loci for replication in an independent Finnish sample of 776 sciatica cases and 18,489 controls from the FINRISK population survey (FINRISK; see URLs). The most significantly associated variant in the meta-analysis (insertion chr9:14344410:I; rs71321981 at 9p22.3, p = 1.30x10^-8^) showed association with sciatica in the replication sample (p = 0.04) (S7 Table). No other replications were identified (S7 Table). The rs190606317 at 6p21.32 as well as rs62100562 at 18q22.3 showing suggestive associations in the meta-analysis (p<1x10^-6^) (S7 Table) had a significant p-value in replication sample (p = 0.006, p = 0.03, respectively), but the effect direction was different and thus was not considered as replicated (S7 Table).

#### Genotype validation

**Fig 4:**
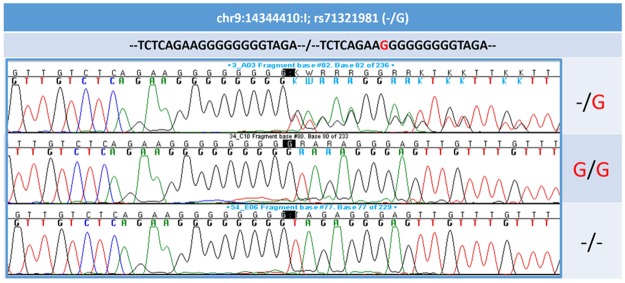
Sequence of the insertion chr9:14344410:I (rs71321981) within the regulatory region of the *NFIB* gene (9p22.3). The chr9:14344410:I (rs71321981) was sequenced in 184 individuals belonging to the YFS and 184 individuals included in the H2000 discovery cohort. Upper panel: heterozygous insertion/frameshift (-/G); Middle panel: homozygous insertion (G/G); Lower panel: wild type (-/-). The nucleotide sequences generated were compared to the reference sequence at 1000 Genomes browser (see URLs).

For validating the imputation accuracy, we sequenced the insertion variant chr9:14344410:I (rs71321981) at 9p22.3 *(NFIB)* in the two discovery cohorts, with 184 individuals (92 cases and 92 controls) in YFS, and 184 individuals (89 cases and 95 controls) in H2000 (Fig 4). The concordance between the sequenced and the imputed genotypes were 88.2% in YFS (imputation quality 0.78; MAF 0.08) and 87.3% in H2000 (imputation quality 0.77; MAF 0.07). We also genotyped rs190200374 and rs80035109 at 15q21.2 (*MYO5A*) in a total of 1686 (152 cases and 1534 controls) and 1642 (154 cases and 1488 controls) individuals in YFS as well as 1405 (82 cases and 1323 controls) and 1392 (99 cases and 1293 controls) individuals in H2000, respectively. For rs190200374, the concordance between the genotyped and imputed genotypes was 96.7% in YFS (imputation quality 0.84; MAF 0.06) and 96.2% in H2000 (imputation quality 0.87; MAF 0.06). For rs80035109, these were 99.6% in YFS (imputation quality 0.97; MAF 0.07) and 98.3% in H2000 (imputation quality 0.97; MAF 0.07). All concordances as well as results from the association analysis using the real genotypes are given in S8 Table. Both rs190200374 and rs80035109 are in strong LD with two other significantly associated variants (rs145901849 and rs117458827) in the *MYO5A* gene region (15q21.2) (r2 ≥ 0.81) (S4 Table). In summary, our additional genotyping assessments were able to validate the accuracy of the imputed genotypes for chr9:14344410:I (9p22.3, *NFIB*), rs190200374 and rs80035109 (15q21.2, *MYO5A)*, with high concordances obtained for all.

## Discussion

The present study is the first GWAS and meta-analysis of sciatica. For lumbar disc degeneration, two genome-wide association analyses have been reported [26, 30]. The strongest association signal (p = 1.30x10^-8^) for sciatica in our present study was obtained for a single base insertion -/G (chr9:14344410:I; rs71321981) within *NFIB* gene at locus 9p22.3. To our knowledge, this locus has not been associated with sciatica earlier. We were able to replicate this association in an independent Finnish population cohort (FINRISK, rs71321981, p = 0.04). Frequency of rs71321981 is about 8% in Finns as well as in other European populations.

The insertion rs71321981 resides in the regulatory region within the first of eight introns of the *NFIB* gene causing a single base extension to the sequence with regard to the reference sequence. It overlaps also a novel antisense gene, *RP11-120J1*.*1*, being intronic or upstream gene variant for that depending on the transcript (Ensemble Variant effect predictor; see URLs).

*NFIB* is a member of the nuclear factor I (NFI) family of evolutionary conserved genes (*NFIA*, *NFIB*, *NFIC* and *NFIX*) encoding sequence-specific DNA-binding proteins, transcription factors, which regulate transcription throughout the development in mammals and in adenoviral replication [31]. NFI transcription factors bind to DNA as homo- and heterodimers [32–34] and control, through promoter and cell-type specific transactivation or repression, a diverse set of tissue-specific and developmentally regulated genes (e.g. in the central nervous system, muscle, and the lungs, as well as in various cell types such as fibroblasts, chondrocytes, osteoblasts, adipocytes) [31, 35–38]. The insertion discovered here is a regulatory region variant located in the C-terminal transcriptional activation and/or repression domain of NFIB [35] (1000 Genomes Project; see URLs).

*In vitro* work has suggested that NFIB plays a role in chondrocyte differentiation [39]. In murine mesenchymal ATDC5 cells, a cell line used as a model for chondrocyte differentiation, the creation of a *NFIB* truncation mutation resulted in lack of the C-terminal transactivation/repression domain, led to an impaired nodule formation, less accumulation of cartilaginous matrices, and reduced expression of a set of marker genes for proliferating chondrocytes, namely *Col2a1*, *Matn-1*, *PTHrP*, and to some extent also of *SOX9* [39]. Other studies have indicated that NFI/NFIB proteins bind to a promoter silencer region of *Matn1* and modulate *Sox9* transactivation in *in vitro* chondrogenesis [40, 41]. NFI has also been demonstrated to function as a positive regulator of *Runx2*-dependent skeletal development and osteoblast function [37].

At 15q21.2, four SNPs; rs145901849 (intronic regulatory region variant), rs117458827 (3’UTR SNP), rs80035109 and rs190200374 (both intronic), were significantly associated with sciatica (p<5x10^-8^). These variants are in strong LD (r^2^≥0.81) within a 200kb region in the *MYO5A* gene. However, we were not able to replicate the 15q21.2 variants in the Finnish replication sample and thus we consider 15q21.2 as a promising locus but needing further replication efforts. In addition, thirteen surrounding SNPs at 15q21.2-15q21.3 harboring the *MYO5A*, *FAM214A*, *ARPP19*, *LYSMD2*, *DMXL2*, and *MYO5C* genes as well as an intergenic region showed suggestive associations (p<1x10^-5^).

Class V myosins (MYO5A, MYO5B, MYO5C; ~50–60% protein sequence identity shared) are an evolutionarily ancient group of molecular motors that mediate actin-dependent organelle trafficking [42, 43]. MYO5A is involved in the intracellular transport of organelles in melanocytes and neuronal cells [44–46], and mutations within it have been associated with rare human syndromes with neurological defects [47–49]. Interestingly, a suggestive association was reported for rs4802666 of *MYH14* (19q13.33) in a GWAS meta-analysis of lumbar disc degeneration [26]. *MYH14* is a member of the same myosin superfamily as *MYO5A* and *MYO5C*; all are expressed in human cell lines derived from bone (Human Protein Atlas; see URLs) and normal skeletal muscle tissue (Gene Cards; see URLs). It has been suggested that myosins may play a role in lumbar disc degeneration through mechanisms that affect multiple tissues, rather than cartilage alone [26]. To sum up, 15q21.2 (*MYO5A*) emerged as an interesting locus but failed to replicate in our replication sample. Further replication efforts in other populations are needed. In this context, it is of note that the *MYO5A* variants discovered appear enriched in the Finnish population (somewhat over 5%) and are rather rare in other European populations (up to 2%) (1000 Genomes Project; see URLs) as discussed in more detail below.

At locus 6p21.32–33, 45 SNPs located within a 1.4 Mb region in the *HLA* gene region (i.e., human major histocompatibility complex) showed suggestive associations with sciatica (p<1x10^-5^). Six of them had a p-value <1x10^-6^, of which four (rs115949512, rs3094014, rs114615271 and rs115688765) were in strong LD (r^2^≥0.97) within or nearby the *HCP5* gene at 6p21.33 and two (rs190606317, rs115488695) were in moderate LD (r^2^≥0.31) within/nearby the *HLA-DRB5* gene at 6p21.32 (S6 Table). We were not able to replicate association between these six lead SNPs and sciatica in our independent Finnish replication sample. The rs190606317 at 6p21.32 had a significant p-value (p = 0.006) in the sample, but the effect direction was different and thus was not considered as replicated (S7 Table).

The *HLA* gene region (6p21.32) has previously been associated with musculoskeletal disorders. Two SNPs (rs2187689 and rs7767277) were associated with lumbar disc degeneration (LDD) in a GWAS meta-analysis in Northern European populations [26], and two variants (rs10947262 and rs7775228) were associated with osteoarthritis (OA) in the GWAS of a Japanese population [27], although this association was not replicated in European or Han Chinese study populations [50, 51]. The OA-associated SNPs (rs7775228, rs10947262) from the Japanese study were not in LD with our six lead variants in the *HLA* gene region, and the LDD-associated variants (rs2187689 and rs7767277) were not included in our GWAS (not included in the 1000 Genomes 1KG pilot data) (S6 Table). It may be noted that neither sciatica, osteoarthritis, nor LDD is considered as an auto-immune disease. However, it has been suggested that there may be pro-inflammatory cytokine activation in herniated lumbar discs [5, 52], and anti-TNF has been used successfully to treat disc herniation-induced sciatica [5, 53]. Accordingly, inflammatory mediator genes are considered to be candidate genes for sciatica, lumbar disc degeneration and osteoarthritis [5, 14, 28, 54, 55].

It is also of note that *COL11A2*, previously associated with magnetic resonance-determined disc signal intensity (with rs2076311 as the lead SNP) and with degenerative lumbar spinal stenosis with radicular pain (the lead SNP rs1799907) [28, 29], lies 622 kb upstream of our rs190606317 at 6p2.32. However, our six lead variants were not in LD with these *COL11A2* variants (rs2076311, rs1799907) (S6 Table), so it seems unlikely that *COL11A2* would directly account for the observed association in the meta-analysis, although we cannot rule out some possible influence of long range effectors such as enhancers.

The primary phenotype in the two discovery GWAS was a physician-diagnosed sciatica. Sciatica is a syndrome involving nerve root impingement or inflammation that has progressed sufficiently to cause neurological symptoms in the areas that are supplied by the affected nerve roots [8]. While there is a range of definitions of sciatica, its specific clinical features—such as low back pain radiating below the knee, presence of numbness or pins and needles in a dermatomal distribution, positive results on a straight leg raise test, and weakness or reflex changes, or both, in a myotomal distribution—are used for diagnoses of sciatica [7, 9]. Further, sciatica is commonly associated with disc disorders such as herniated disk or spinal stenosis [9], but still in many cases with clinical symptoms of sciatica, no lumbar disc herniation is present on images [9, 56, 57]

In YFS, the physician-diagnosed sciatica cases were self-reported in an on-site examination and represented manifestations of sciatica in a relatively young population. In H2000, sciatica was diagnosed by a field physician, if the subject had a history of low back pain radiating down to the leg, and either positive clinical findings or a history of lumbar disc herniation that had previously been confirmed by imagining or required surgery. Finally, in the replication population (FINRISK, the largest of the study populations), the sciatica phenotype was based on ICD diagnoses available at the Finnish Hospital Discharge Register, thus likely to represent the severe cases requiring hospitalization. Collectively, while the phenotypes across the three study cohorts were not identical, they were all representative of sciatica. Therefore, we see that the study managed to capture representation from the whole sciatica spectrum. Furthermore, in all study populations, the diagnoses were made by Finnish physicians according to general medical practice in Finland, a country with high-level and uniform medical education and well organized health services. Obviously, some residual differences between the diagnoses and study populations may partly explain why only one of the loci (9p22.3) was replicated in the FINRISK population.

This study is based on three representative Finnish population samples representing a genetic isolate, where certain rare and low frequency variants are enriched due to population history (small founder population, several bottle neck events and genetic drift). Each of the five lead SNPs in the two loci (9p22.3, 15q21.2) has a relatively low frequency (<8%) in the general population (1000 Genomes Project; see URLs). The insertion chr9:14344410:I at 9p22.3 (*NFIB*) has similar frequencies in the Finnish (7%) and other European populations (8%) as well as in American (6%) and South Asian populations (8%) (1000 Genomes Project; see URLs). In African population, the frequency of rs71321981 is even higher (24%), but in East Asian population this variant is absent (0%). Most of these minor allele frequencies would allow replication; we therefore conclude that further replication efforts are warranted. For 15q21.2, however, all four *MYO5A* variants were 3–6 times more frequent in our representative Finnish samples (6–7%) than in other European populations (0–2%) and in African, American, East and South Asian populations they were absent (Table 2; S5 Table; 1000 Genomes Project; see URLs). The differences in allele frequencies likely indicates a past bottleneck events and genetic drift in the Finnish population. In all, our data are in line with previous studies illustrating the high utility of population isolate and the dense genotype imputation based on representative data from multiple populations, in search for low frequency variants associated with complex human diseases [21, 58–60]. The five relatively low frequency variants associated with sciatica in this study, especially the differentiated variants in 15q21.2, would likely not have been identified without inclusion of Finnish individuals in the 1000 Genomes reference panel. Furthermore, the identification and replication of the differentiated variants would require much larger sample sizes in more mixed population.

In summary, we conducted the first GWAS meta-analysis of sciatica and identified a single base insertion at locus 9p22.3 (*NFIB*) associated with sciatica at genome-wide significance and replicated in an independent Finnish population sample. The insertion is within a regulation region of the transcription factor *NFIB*, which has been shown to be involved in chondrocyte differentiation and osteoblast function, thus making this gene and the insertion functionally interesting for sciatica. In addition, we identified four variants associated with sciatica at the locus 15q21.2 *(MYO5A*), which was promising but not replicated. Both loci merit further investigation and replication studies. As the first GWAS of sciatica, this study may serve as a starting point for further studies and shed light to the genetic susceptibility factors of sciatica.

## Materials and Methods

### Study populations and phenotypes

This study was carried out in accordance with the recommendations of the Declaration of Helsinki. All participants of studies have given written informed consent. Studies were approved by the local research ethic committees: Ethics Committee of the National Public Health Institute for the Health 2000 Study, Ethics Committee of the Hospital District of Southwest Finland for the Young Finns Study, and Ethics Committee of Helsinki and Uusimaa Hospital for the FINRISK Study.

Genome-wide association studies of sciatica were carried out in two Finnish population-based cohorts, the Young Finns Study, (YFS; 180 sciatica cases and 1,840 controls) and the Health 2000 Study (H2000; 111 sciatica cases and 1,831 controls) (Fig 1; S1 Table; S1 Text). The primary phenotype analyzed was a physician-diagnosed sciatica, with diagnosis based on specific symptoms and clinical findings according to general medical practice in Finland. In the YFS, information on physician-diagnosed sciatica was inquired during on-site examinations using a self-administered questionnaire (“Do you currently have or have you had a long-term disease diagnosed by a physician, such as sciatica?”). In the H2000, participants attended a comprehensive health examination, with a physical examination of the musculoskeletal system performed by a field physician. The diagnosis of sciatica was based on the presence of chronic (>3 months) low back pain radiating down to the leg and either clinical findings of lumbar nerve root compression or a history of lumbar disc herniation that had been previously verified by imaging or required surgery (S1 Text). Demographics of the population samples are given in Table 1 (see also S1 Text).

Replication analyses were carried out in an independent Finnish population sample consisting of four independent cross-sectional population surveys (carried out in years 1992, 1997, 2002, and 2007) of the FINRISK Study [61] (Table 1; S1 Text; see URLs). The FINRISK Study populations have been linked to the Finnish Hospital Discharge Register (currently the Finnish Care Register for Health Care) (see URLs), which provides personal identification code-based individual diagnoses (WHO ICD codes) at discharge. For the replication study, those diagnosed with one of the ICD-codes selected *a priori* by two experts on musculoskeletal diseases (EVJ and MH) as relevant for sciatica or sciatic syndrome (ICD8 353, 728.8; ICD9 724.3, 722.1, 722.10, 722.5, 722.52, 355.0; ICD10 M54.3, M51.1, M54.1, M54.4) were included as cases (amounting to 776 sciatica cases and 18,489 controls) (see URLs; S1 Text).

### Genome-wide scans and imputation

Genotypes for both YFS and H2000 study populations were pre-existing, determined at the Wellcome Trust Sanger Institute (UK) using custom-generated Illumina Human Map 670K array for YFS and 610K BeadChip for H2000 study. Prior to genome-wide association analysis, quality control was performed independently in both two cohorts. Poor quality markers (those with genotyping failure >5% of samples) and poor quality DNA samples (those with genotyping failure>5% of markers) were removed. Moreover, markers with low minor allele frequency (MAF<0.01 in YFS and MAF<0.02 in H2000), Mendelian errors, or those violating the Hardy-Weinberg equilibrium (HWE ≤1x10^-6^ in YFS and in H2000) were excluded. Likewise, samples with gender inconsistency or cryptic relatedness (PI_HAT>0.2) and samples with excessive genome-wide heterozygosity (indicative of sample contamination) were removed. IBD sharing was also computed for the combined dataset, and duplicates and close relatives (PI_hat>0.4) were removed from the analyses. The genotype imputation data used were generated from cleaned data in both cohorts using IMPUTE2–program [62] and was based on the 1000 Genomes imputation reference in NCBI build 37, where the 1000 Genomes files were from March 2012 release for YFS and April 2012 release for H2000 (see URLs). The 1000 Genomes imputation reference includes Finnish imputation reference (FIN; see URLs). Quality control for imputed markers was conducted separately in both studies; markers with MAF<0.02 or imputation quality <0.7 were excluded. Genotyping, imputation and quality control measures are summarized in S1 Table.

### Genome-wide association analyses and meta-analysis

The genome-wide scan data was analyzed for associations between genetic variants and sciatica separately in YFS and H2000 studies (Fig 1). Multidimensional Scaling was done for genetic data of both studies using PLINK v. 1.07 [63]. Genome-wide association analyses were performed for directly genotyped and imputed variants. Both studies included as covariates age, sex and the first seven principal components from the genetic data to correct possible population stratification. Frequentist/case-control-test, assuming an additive genetic model, was performed using SNPTEST v2 for both genotyped and imputed markers [64]. To combine the effect estimates from both studies, a fixed-effects meta-analysis was conducted for sciatica using GWAMA [65]. The presence of heterogeneity across studies was investigated with Cochran’s Q (weighted sum of squares) test and *I*^*2*^ statistic (percentage of true heterogeneity to total observed variation) [66]. Only good quality autosomal markers passing the following criteria: imputation informativeness >0.7, and no heterogeneity in the effect sizes for the SNP between cohorts (Cochran‘s Q statistic P-value<1x10^-5^), were included in further evaluations. The genome-wide inflation factor was measured in the individual GWAS and the GWAS meta-analysis by genomic control statistic [67]. There was no evidence for population stratification at the study level (genomic inflation factor; YFS λ_GC_ = 1.001 and H2000 λ_GC_ = 1.016) or at the meta-analysis level (λ_GC_ = 0.993). Test statistics of both GWAS were corrected by using a genomic inflation factors. The Quantile-Quantile and Manhattan plots were created using R-2.11 (see URLs) to visualize genome-wide association results. Regional plots of association were generated using LocusZoom [68] (see URLs). The genomic positions indicated throughout this study are based on NCBI human genome build 37 (see URLs). GWAS, GWAS meta-analysis, and quality control measures are summarized in S1 Table.

### Replication analysis

Variants with p-value < 1 x 10^−6^ in the genome-wide meta-analysis were selected for replication. Variant was considered replicated if it reached significance of p<0.05 and was consistent in terms of risk allele.

A large Finnish replication cohort, the FINRISK Study, was used for replication. Genotypes for FINRISK were pre-existing and, due to the large number of participants (close to 21 000), genotyping was performed in multiple batches/subpopulations using several standard genotyping arrays including: Sanger CoreExome batch1, Illumina HumanCoreExome Sanger CoreExome batch2, Illumina HumanCoreExome Broad CoreExome batch1, Illumina HumanCoreExome PredictCVD, Illumina OmniX Corogene, Illumina 610K SUMMIT, Illumina OmniX MIGEN–and Affy 6. Genotyping quality was examined by a detailed QC procedure consisting of success rate checks, duplicated water controls and Hardy Weinberg Equilibrium (HWE) testing. The genotype imputation data used was generated from cleaned data using IMPUTE2–program and was based on the 1000 Genomes imputation reference panel.

Frequentist association test assuming an additive genetic model was performed using SNPTEST v2 for selected variants [64].

### Genotype validation

To validate imputation accuracy, the imputed variant chr9:14344410:I (rs71321981) at 9p22.3 was genotyped by direct sequencing using standard methods as described elsewhere [69]. The chromatograms were analyzed manually, and the corresponding nucleotide sequences were compared to the reference sequence at 1000 Genomes browser (1000 Genomes Project; see URLs). The primer sequences are available from the authors on request.

In addition, imputed variants rs190200374 and rs80035109 at 15q21.2 were genotyped using a TaqMan^®^ chemistry-based PCR platform (Open Array^™^ system) and custom-made TaqMan^®^ SNP Genotyping assays (Applied Biosystems). The allelic calling analysis was performed using TaqMan Genotyper v1.3 software and OpenArray^™^ SNP Genotyping Analysing software. For quality control, two independent readers interpreted the results. Random selection of all samples (about 5% in H2000 and 12% in YFS) was re-genotyped. No discrepancies were discovered in the replicate tests for the variants.

Concordances between the genotyped and imputed SNPs were calculated using threshold 0.7 for converting probabilistic genotypes of imputed SNPs to hard calls.

### Web Resources

The URLs for data presented herein are as follows:

GWAMA, http://www.well.ox.ac.uk/gwama/

IMPUTE2, http://mathgen.stats.ox.ac.uk/impute/impute_v2.html

SNPTEST, https://mathgen.stats.ox.ac.uk/genetics_software/snptest/old/snptest_v2.1.1.html

LocusZoom, http://csg.sph.umich.edu/locuszoom/

R, http://www.r-project.org/

1000 Genomes Project, http://www.1000genomes.org/

Ensemble Variant Effect Predictor: http://www.ensembl.org/info/docs/tools/vep/index.html

Human Protein Atlas, http://www.proteinatlas.org/

Gene Cards, http://www.genecards.org/

Cardiovascular Risk in Young Finns Study, http://youngfinnsstudy.utu.fi/

Health 2000 Study, http://www.nationalbiobanks.fi/index.php/studies2/8-health2000

FINRISK, http://www.nationalbiobanks.fi/index.php/studies2/7-finrisk

Finnish Care Register for Health Care, https://www.thl.fi/fi/web/thlfi-en/statistics/information-on-statistics/register-descriptions/care-register-for-health-care

## Supporting Information

S1 Appendix Raw result data of the meta-analysis (with variants with p < 0.05). (TXT)

S2 Appendix Raw result data of the meta-analysis (with variants with p < 0.001). (TXT)

S1 Fig Quantile-quantile plot of observed against expected p-values for the genome-wide meta-analysis of sciatica. (TIF)

S2 Fig Manhattan plot for the Young Finns Study GWAS of sciatica. Results are adjusted for the first seven principal components of genetic data and for sex and age. Variants in red have p-value below genome-wide significance level (p < 5x10^-8^).
(TIFF)

S3 Fig Quantile-quantile plot of observed against expected p-values for the Young Finns Study GWAS of sciatica. Results are adjusted for the first seven principal components of genetic data and for sex and age.
(TIF)

S4 Fig Manhattan plot for the Health 2000 GWAS of sciatica. Results are adjusted for the first seven principal components of genetic data, and for sex and age.
(TIF)

S5 Fig Quantile-quantile plot of observed against expected p-values for the Health 2000 GWAS of sciatica. Results are adjusted for the first seven principal components of genetic data, and for sex and age.
(TIF)

S1 Table Study analysis methods. Methods used in genotyping, imputation, genome-wide association study and meta-analysis.
(DOCX)

S2 Table Results of the genome-wide meta-analysis of sciatica showing variants with p < 1x10-5. (DOCX)

S3 Table Variants associated with sciatica in the meta-analysis (p < 1x10^-6^) with effect allele frequencies, imputation quality and p-values in two discovery GWAS. (DOCX)

S4 Table LD estimates from the YFS (upper diagonal in green) and H2000 (lower diagonal in yellow) for SNPs with p<5x10^-8^ within the locus 15q21.2. (DOCX)

S5 Table Minor allele frequencies for the five most promising variants in the Finnish and other European populations. (DOCX)

S6 Table LD estimates from the YFS (upper diagonal in green) and H2000 (lower diagonal in yellow) for the SNPs in the HLA region within the locus 6p21.32. (DOCX)

S7 Table The most promising variants in the GWAS meta-analysis of sciatica (p<1x10^-6^) as tested for replication in a Finnish population-based cohort (FINRISK). (DOCX)

S8 Table Genotype validations with three variants showing genome-wide significant association in the GWAS meta-analysis subsequently genotyped in individuals of the Young Finns Study and Health 2000 discovery cohorts. (DOCX)

S1 Text Study populations and phenotypes. (DOCX)

S2 Text Supplementary References. (DOCX)
