# Supplementary figures and images for: Genome-Wide Meta-Analysis of Sciatica in Finnish Population
Source: PLoS One. 2016 Oct 20;11(10):e0163877. doi: 10.1371/journal.pone.0163877 (PMC5072673; doi:10.1371/journal.pone.0163877)

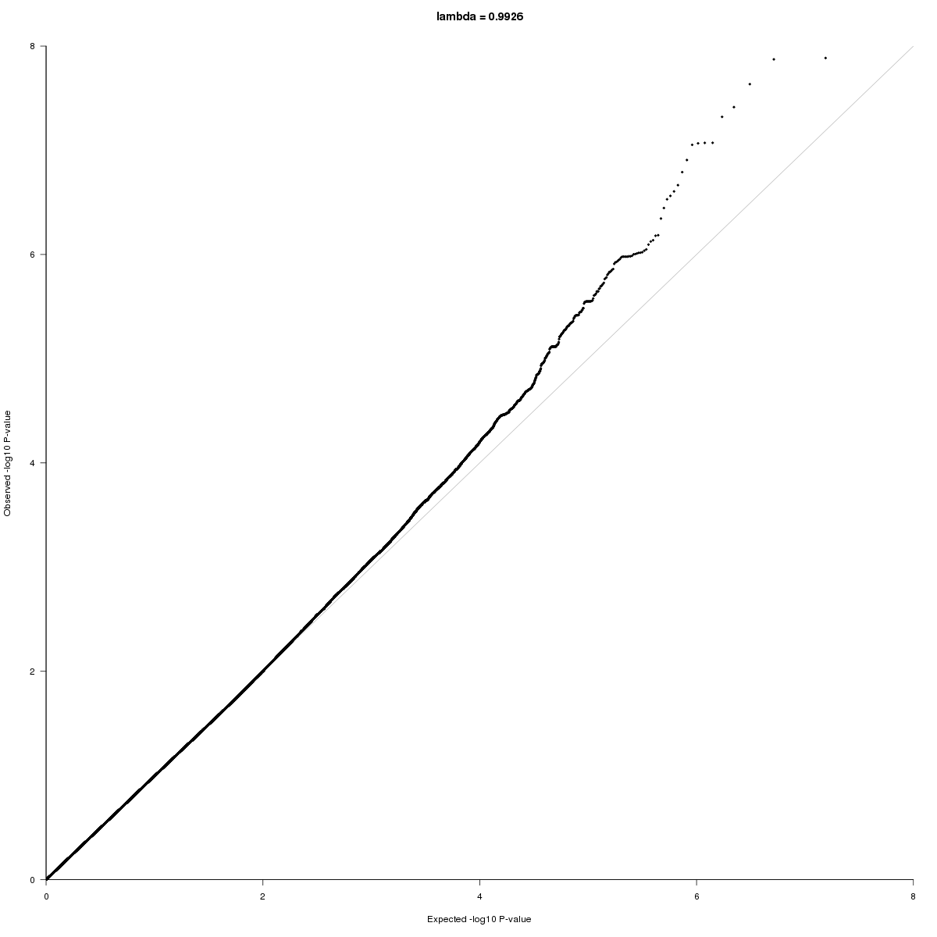

Supplement: S1 Fig — (TIF) [file pone.0163877.s003.tif]

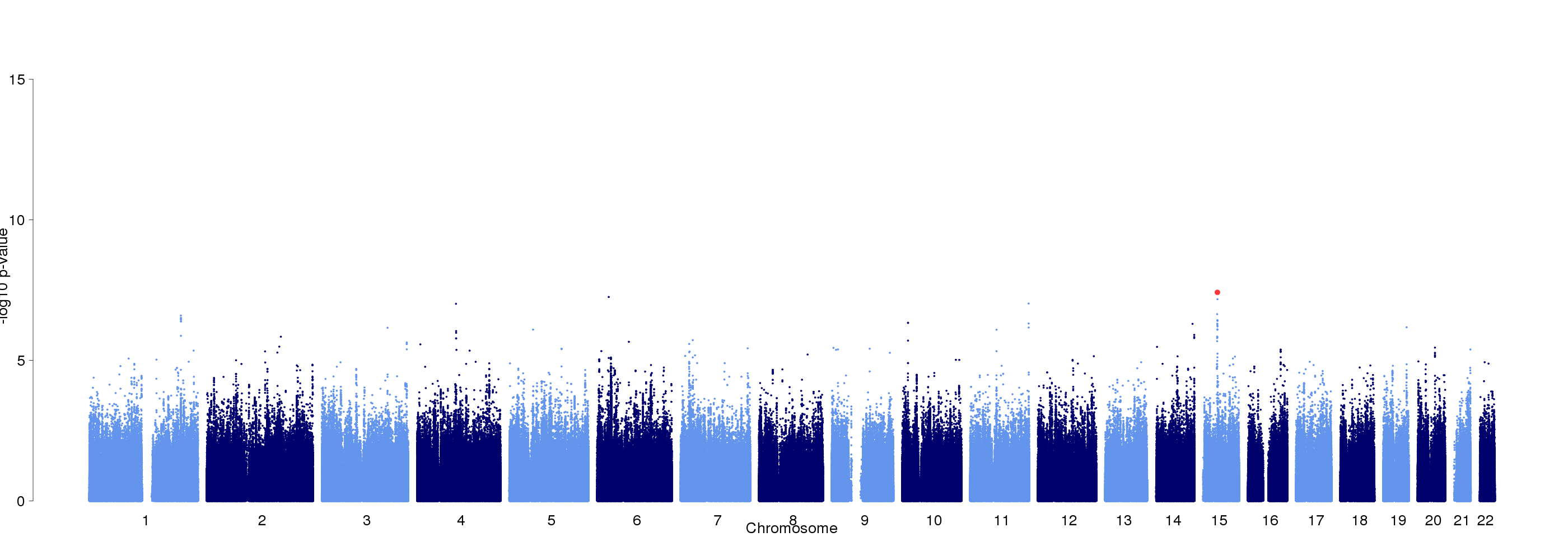

Supplement: S2 Fig — Results are adjusted for the first seven principal components of genetic data and for sex and age. Variants in red have p-value below genome-wide significance level (p < 5x10^-8^). (TIFF) [file pone.0163877.s004.tiff]

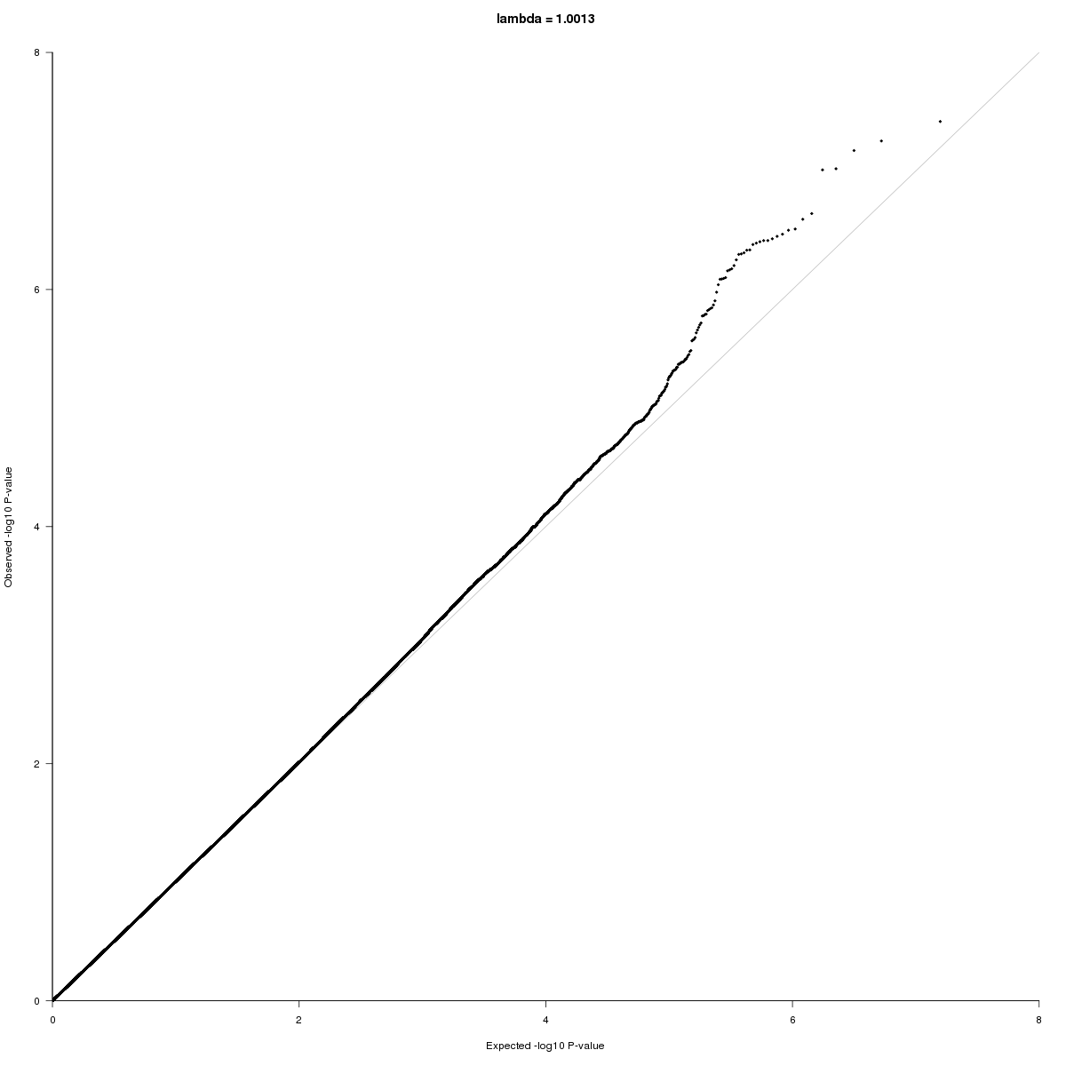

Supplement: S3 Fig — Results are adjusted for the first seven principal components of genetic data and for sex and age. (TIF) [file pone.0163877.s005.tif]

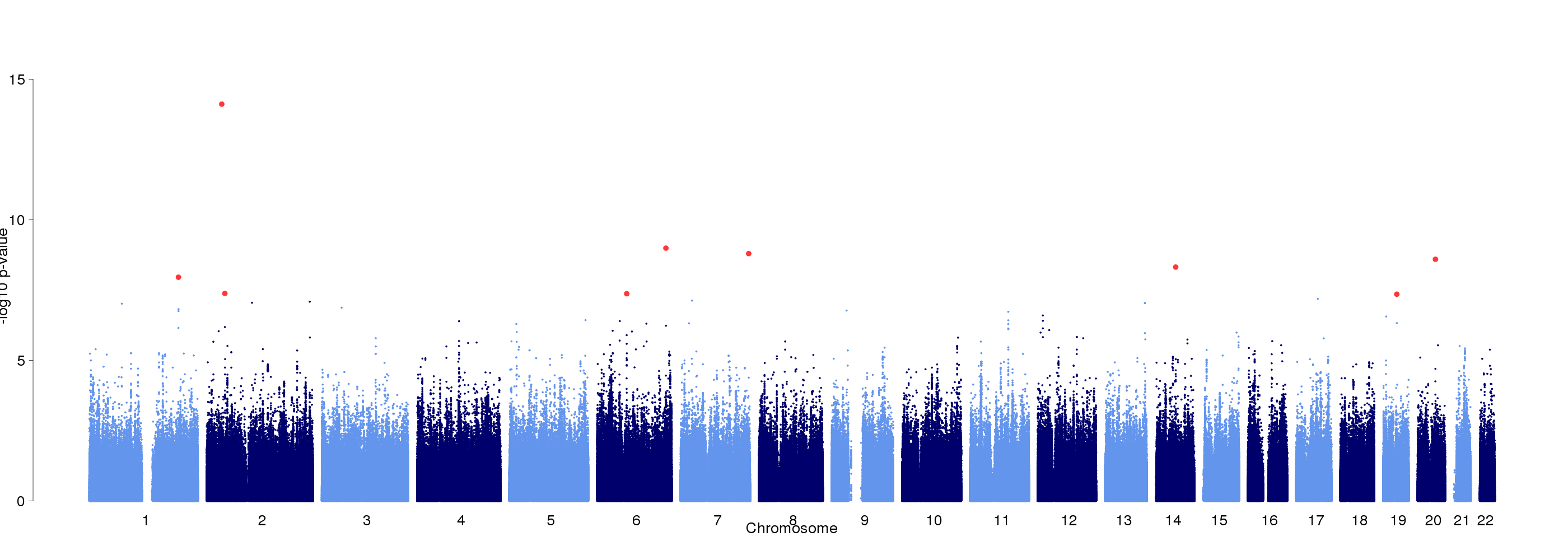

Supplement: S4 Fig — Results are adjusted for the first seven principal components of genetic data, and for sex and age. (TIF) [file pone.0163877.s006.tif]

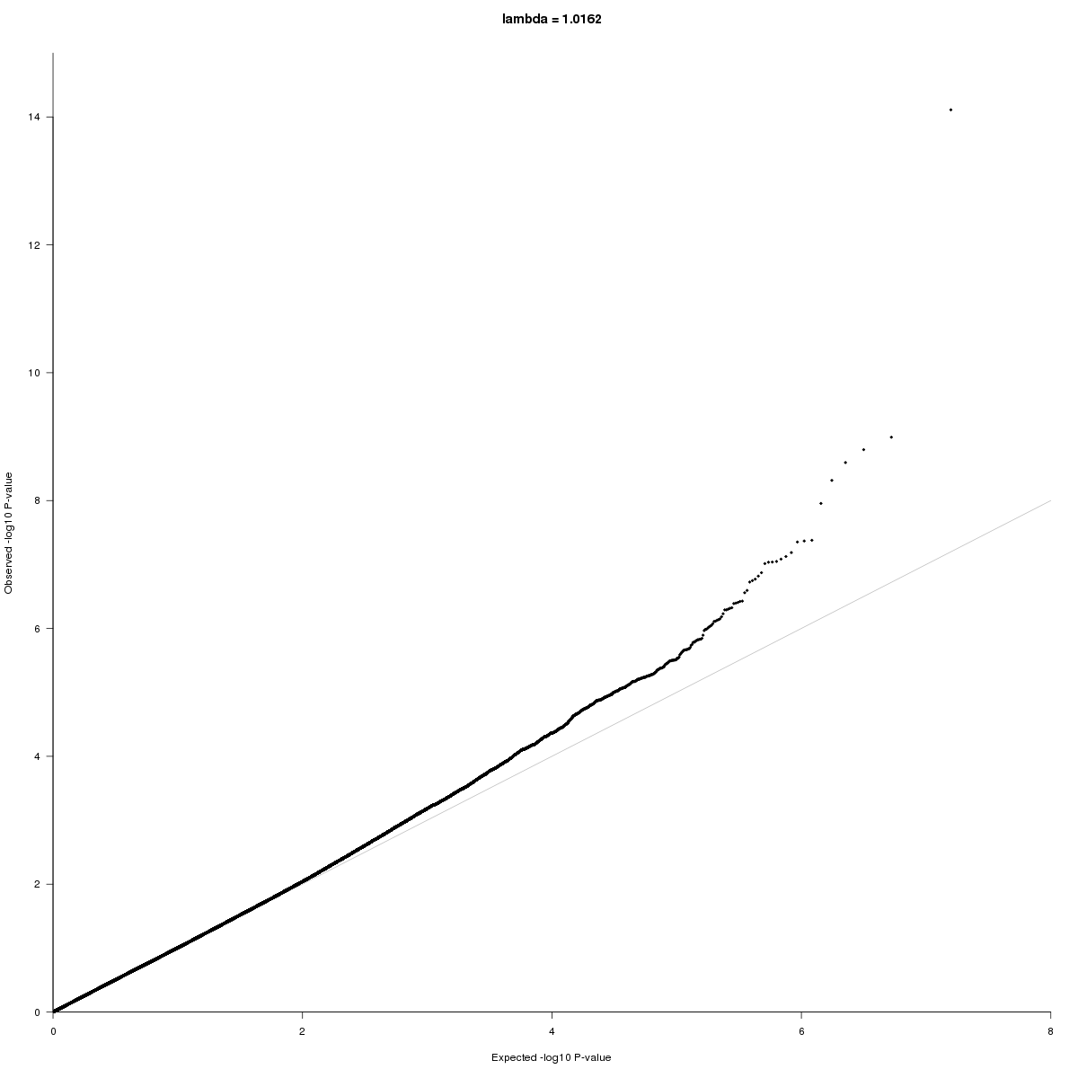

Supplement: S5 Fig — Results are adjusted for the first seven principal components of genetic data, and for sex and age. (TIF) [file pone.0163877.s007.tif]
