# Supplementary material for: Genome-Wide Meta-Analysis of Sciatica in Finnish Population
Source: PLoS One. 2016 Oct 20;11(10):e0163877. doi: 10.1371/journal.pone.0163877 (PMC5072673; doi:10.1371/journal.pone.0163877)
Supplement: S1 Table — Methods used in genotyping, imputation, genome-wide association study and meta-analysis. (DOCX) [file pone.0163877.s008.docx]

|  | **Young Finns Study** | **Health 2000 Study** |
| --- | --- | --- |
| **Genotyping** | | |
| **Chip** | Illumina Human Map 670K | Illumina Human Map 610K |
| **Total number of SNPs after QC** | 546 674 | 592 361 |
| **GWA -analysis program** | PLINK  (logistic regression analysis) | PLINK  (logistic regression analysis) |
| **Imputation** | | |
| **Imputation software** | IMPUTE | IMPUTE |
| **Imputation reference** | 1000 Genomes imputation reference  (March 2012 release) | 1000 Genomes imputation reference  (April 2012 release) |
| **Genome build** | NCBI build 37 | NCBI build 37 |
| **Total number of SNPs after imputation and QC** | 7.9 million | 7.9 million |
| **GWA –analysis program** | SNPTEST v 2.4.0  (case-control test) | SNPTEST v 2.4.0  (case-control test) |
| **Meta-analysis** | | |
| **Total number of SNPs after meta-analysis and QC (imput quality>0.7)** | 7.7 million | |
| **Meta-analysis program** | GWAMA  (fixed-effects model) | GWAMA  (fixed-effects model) |

## Supplementary Table S1. Study analysis methods.

Methods used in genotyping, imputation, genome-wide association study and meta-analysis.
