# Supplementary material for: Genome-Wide Meta-Analysis of Sciatica in Finnish Population
Source: PLoS One. 2016 Oct 20;11(10):e0163877. doi: 10.1371/journal.pone.0163877 (PMC5072673; doi:10.1371/journal.pone.0163877)
Supplement: S2 Table — (DOCX) [file pone.0163877.s009.docx]

## Supplementary Table S2. Results of the genome-wide meta-analysis of sciatica showing variants with p < 1x10^-5^.

| **SNP** | **Chr** | **Position^$^** | **Gene** | **Effect allele** | **Other allele** | **Effect allele freq.** | **N** | **P_het_** | ***I^2^*** | **OR (95% CI)** | **P value** |
| --- | --- | --- | --- | --- | --- | --- | --- | --- | --- | --- | --- |
| chr9:14344410:I | 9p22.3 | 14344410 | NFIB | AG | A | 0.08 | 3960 | 0.82 | 0 | 3.05  (2.08-4.49) | 1.30E-08 |
| rs145901849 | 15q21.2 | 52640539 | MYO5A | T | C | 0.06 | 3961 | 0.11 | 0.61 | 3.04  (2.07-4.45) | 1.34E-08 |
| rs80035109 | 15q21.2 | 52665890 | MYO5A | C | T | 0.07 | 3962 | 0.32 | 0 | 2.65  (1.88-3.72) | 2.32E-08 |
| rs190200374 | 15q21.2 | 52811959 | MYO5A | T | G | 0.06 | 3962 | 0.10 | 0.63 | 2.89  (1.98-4.21) | 3.85E-08 |
| rs117458827 | 15q21.2 | 52600066 | MYO5A | A | G | 0.07 | 3960 | 0.35 | 0 | 2.53  (1.81-3.53 | 4.78E-08 |
| rs149430802 | 15q21.3 | 52948838 | FAM214A | T | C | 0.07 | 3961 | 0.20 | 0.38 | 2.56  (1.82-3.61) | 8.48E-08 |
| rs117288416 | 15q21.3 | 52957840 | FAM214A | T | C | 0.07 | 3961 | 0.20 | 0.38 | 2.56  (1.82-3.61) | 8.49E-08 |
| chr15:52604566:I | 15q21.2 | 52604566 | MYO5A | CT | C | 0.07 | 3960 | 0.39 | 0 | 2.70  (1.88-3.89) | 8.58E-08 |
| rs143229532 | 15q21.3 | 52928933 | FAM214A | C | T | 0.07 | 3961 | 0.20 | 0.39 | 2.58  (1.82-3.64) | 8.86E-08 |
| rs117930495 | 15q21.2 | 52743583 | MYO5A | C | T | 0.07 | 3961 | 0.21 | 0.35 | 2.48  (1.77-3.46) | 1.24E-07 |
| rs80026449 | 11q12.2 | 60317643 | - | A | G | 0.03 | 3962 | 0.19 | 0.41 | 5.50 (2.91-10.40) | 1.62E-07 |
| rs183165962 | 15q21.2 | 52514666 | MYO5C | A | G | 0.07 | 3962 | 0.15 | 0.51 | 2.57  (1.80-3.67) | 2.16E-07 |
| chr15:52852285:I | 15q21.2 | 52852285 | ARPP19 | TA | T | 0.07 | 3961 | 0.19 | 0.43 | 2.52  (1.77-3.58 | 2.48E-07 |
| rs190606317 | 6p21.32 | 32508053 | - | A | G | 0.15 | 3960 | 0.11 | 0.61 | 2.18  (1.62-2.93) | 2.74E-07 |
| rs73937196 | 18p11.31 | 5636910 | - | T | C | 0.05 | 3962 | 0.77 | 0 | 2.91  (1.93-4.37) | 2.95E-07 |
| rs115488695 | 6p21.32 | 32490036 | HLA-DRB5 | T | C | 0.18 | 3960 | 0.85 | 0 | 1.98  (1.52-2.58) | 3.58E-07 |
| rs58509608 | 18p11.31 | 5640484 | - | C | T | 0.05 | 3961 | 0.71 | 0 | 2.81  (1.88-4.21) | 4.52E-07 |
| rs186767095 | 15q21.2 | 52388742 | - | A | T | 0.06 | 3960 | 0.08 | 0.68 | 2.66  (1.81-3.92) | 6.53E-07 |
| rs62100562 | 18q22.3 | 71537484 | - | T | G | 0.02 | 3961 | 0.21 | 0.35 | 5.37 (2.77-10.42) | 6.61E-07 |
| rs115949512 | 6p21.33 | 31430721 | HCP5 | G | A | 0.14 | 3961 | 0.29 | 0.09 | 1.88  (1.47-2.42) | 7.30E-07 |
| rs117146116 | 9p21.1 | 28313102 | LINGO2 | C | T | 0.03 | 3961 | 0.58 | 0 | 4.45  (2.46-8.03) | 7.50E-07 |
| rs3094014 | 6p21.33 | 31433558 | HCP5 | A | G | 0.13 | 3962 | 0.22 | 0.33 | 1.89  (1.47-2.42) | 8.03E-07 |
| rs2241921 | 11q12.2 | 60164302 | MS4A14 | T | C | 0.64 | 3962 | 0.52 | 0 | 1.56  (1.31-1.87) | 8.93E-07 |
| rs6591578 | 11q12.2 | 60158649 | MS4A7 | A | G | 0.64 | 3962 | 0.56 | 0 | 1.57  (1.31-1.88) | 9.19E-07 |
| rs114615271 | 6p21.33 | 31434198 | - | C | T | 0.13 | 3960 | 0.22 | 0.33 | 1.89  (1.47-2.44) | 9.51E-07 |
| rs10145254 | 14q13.1 | 34534344 | - | T | C | 0.03 | 3960 | 0.41 | 0 | 4.78  (2.56-8.92) | 9.62E-07 |
| rs115688765 | 6p21.33 | 31433831 | HCP5 | G | A | 0.13 | 3962 | 0.23 | 0.32 | 1.89  (1.46-2.43) | 9.79E-07 |
| rs10792269 | 11q12.2 | 60117126 | - | A | G | 0.64 | 3962 | 0.77 | 0 | 1.56  (1.31-1.87) | 9.91E-07 |
| rs77310140 | 4p15.1 | 35532585 | - | G | A | 0.04 | 3962 | 0.61 | 0 | 4.36  (2.42-7.86) | 9.97E-07 |
| rs12880515 | 14q21.1 | 40379061 | - | T | C | 0.14 | 3960 | 0.22 | 0.32 | 1.89  (1.47-2.45) | 1.03E-06 |
| rs192797643 | 15q21.3 | 52946250 | FAM214A | C | T | 0.07 | 3961 | 0.34 | 0 | 2.33  (1.66-3.27) | 1.04E-06 |
| rs150141424 | 10q24.32 | 104234622 | TMEM180 | T | C | 0.03 | 3961 | 0.77 | 0 | 3.07  (1.96-4.81) | 1.04E-06 |
| rs2233253 | 11q12.2 | 60157166 | MS4A7 | T | C | 0.64 | 3962 | 0.53 | 0 | 1.57  (1.31-1.87) | 1.05E-06 |
| rs2233252 | 11q12.2 | 60157107 | MS4A7 | G | T | 0.64 | 3962 | 0.53 | 0 | 1.57  1.31-1.87) | 1.05E-06 |
| rs2014121 | 11q12.2 | 60156297 | MS4A7 | C | T | 0.64 | 3961 | 0.53 | 0 | 1.57  (1.31-1.87) | 1.05E-06 |
| rs7935082 | 11q12.2 | 60155000 | MS4A7 | C | T | 0.64 | 3961 | 0.53 | 0 | 1.57  (1.31-1.87) | 1.05E-06 |
| rs950803 | 11q12.2 | 60152563 | MS4A7 | A | T | 0.64 | 3961 | 0.53 | 0 | 1.57  (1.31-1.87) | 1.05E-06 |
| rs9834633 | 3p22.3 | 34334484 | - | A | G | 0.09 | 3961 | 0.51 | 0 | 0.45  (0.33-0.62) | 1.07E-06 |
| rs2241920 | 11q12.2 | 60164204 | MS4A14 | A | G | 0.64 | 3962 | 0.53 | 0 | 1.56  (1.31-1.87) | 1.11E-06 |
| rs183940360 | 10p13 | 13380620 | SEPHS1 | G | C | 0.02 | 3961 | 0.06 | 0.72 | 4.43  (2.43-8.06) | 1.13E-06 |
| rs77798076 | 9p24.1 | 4655323 | SPATA6L | G | A | 0.06 | 3960 | 0.77 | 0 | 2.67  (1.80-3.96) | 1.16E-06 |
| rs1822366 | 11q12.2 | 60120539 | - | G | T | 0.64 | 3962 | 0.77 | 0 | 1.57  (1.31-1.87) | 1.18E-06 |
| rs147919067 | 10p13 | 13393866 | - | G | C | 0.02 | 3960 | 0.06 | 0.72 | 4.44  (2.43-8.09) | 1.19E-06 |
| rs12798266 | 11q12.2 | 60118851 | - | A | G | 0.65 | 3961 | 0.77 | 0 | 1.56  (1.31-1.87) | 1.23E-06 |
| rs80055220 | 11q12.2 | 60275495 | - | T | C | 0.03 | 3961 | 0.18 | 0.43 | 4.45  (2.43-8.15) | 1.40E-06 |
| rs116105128 | 6p21.32 | 32511456 | - | C | G | 0.11 | 3960 | 0.49 | 0 | 2.20  (1.60-3.03) | 1.47E-06 |
| rs12886276 | 14q21.1 | 40373048 | - | G | A | 0.14 | 3960 | 0.23 | 0.30 | 1.87  (1.45-2.42) | 1.47E-06 |
| rs11230288 | 11q12.2 | 60128968 | - | A | G | 0.64 | 3962 | 0.76 | 0 | 1.56  (1.30-1.87) | 1.53E-06 |
| rs4528348 | 11q12.2 | 60129505 | - | G | C | 0.64 | 3961 | 0.76 | 0 | 1.56  (1.30-1.87) | 1.57E-06 |
| rs183822499 | 10q13 | 13384481 | SEPHS1 | A | G | 0.03 | 3961 | 0.15 | 0.51 | 3.28  (2.02-5.33) | 1.66E-06 |
| rs143451380 | 6p21.32 | 32489571 | HLA-DRB5 | G | C | 0.16 | 3960 | 0.41 | 0 | 1.97  (1.49-2.60) | 1.69E-06 |
| rs114043444 | 6p21.32 | 32520595 | HLA-DRB6/HLA-DRB4 | A | G | 0.25 | 3960 | 0.48 | 0 | 1.75  (1.39-2.20) | 1.72E-06 |
| chr11:60129828:D | 11q12.2 | 60129828 | - | T | TTG | 0.64 | 3960 | 0.78 | 0 | 1.56  (1.30-1.87) | 1.96E-06 |
| rs73291863 | 14q23.1 | 58250665 | SLC35F4 | A | G | 0.03 | 3961 | 0.08 | 0.67 | 3.32  (2.03-5.45) | 2.01E-06 |
| rs3825020 | 11q12.2 | 60184191 | MS4A14 | A | G | 0.59 | 3962 | 0.90 | 0 | 1.54  (1.29-1.83) | 2.11E-06 |
| rs955019 | 11q12.2 | 60174250 | MS4A14 | T | C | 0.59 | 3961 | 0.90 | 0 | 1.54  (1.29-1.84) | 2.14E-06 |
| rs4470244 | 18p11.31 | 5676759 | - | A | G | 0.07 | 3962 | 0.97 | 0 | 2.27  (1.62-3.19) | 2.26E-06 |
| rs12457186 | 18p11.31 | 5670735 | - | C | T | 0.07 | 3962 | 0.97 | 0 | 2.27  (1.62-3.19) | 2.26E-06 |
| rs7944025 | 11q12.2 | 60142538 | - | A | C | 0.64 | 3960 | 0.72 | 0 | 1.55  (1.29-1.86) | 2.26E-06 |
| rs189906729 | 9p24.1 | 4686426 | CDC37L1 | T | C | 0.03 | 3960 | 0.10 | 0.63 | 3.91  (2.22-6.89) | 2.46E-06 |
| chr6:31310025:I | 6p21.33 | 31310025 | - | CT | C | 0.13 | 3961 | 0.83 | 0 | 2.03  (1.51-2.73) | 2.47E-06 |
| rs7926219 | 11q12.2 | 60180335 | MS4A14 | T | C | 0.59 | 3961 | 0.90 | 0 | 1.53  (1.28-1.83) | 2.64E-06 |
| rs184095703 | 15q21.2 | 52038741 | LYSMD2 | T | C | 0.07 | 3961 | 0.10 | 0.64 | 2.24  (1.6-3.13) | 2.76E-06 |
| rs16948608 | 18p11.31 | 5669955 | - | A | T | 0.07 | 3960 | 0.97 | 0 | 2.27  (1.61-3.2) | 2.79E-06 |
| chr18:5669954:D | 18p11.31 | 5669954 | - | A | AT | 0.07 | 3962 | 0.97 | 0 | 2.27  (1.61-3.2) | 2.80E-06 |
| rs11876234 | 18p11.31 | 5675566 | - | A | G | 0.07 | 3962 | 0.97 | 0 | 2.27  (1.61-3.2) | 2.82E-06 |
| rs59892835 | 18p11.31 | 5674741 | - | T | C | 0.07 | 3962 | 0.97 | 0 | 2.27  (1.61-3.2) | 2.82E-06 |
| rs875586 | 18p11.31 | 5674268 | - | A | C | 0.07 | 3962 | 0.97 | 0 | 2.27  (1.61-3.2) | 2.82E-06 |
| rs72634391 | 18p11.31 | 5673204 | - | C | T | 0.07 | 3962 | 0.97 | 0 | 2.27  (1.61-3.2) | 2.82E-06 |
| rs72634390 | 18p11.31 | 5672053 | - | C | G | 0.07 | 3962 | 0.97 | 0 | 2.27  (1.61-3.2) | 2.82E-06 |
| rs12457942 | 18p11.31 | 5671895 | - | G | T | 0.07 | 3962 | 0.97 | 0 | 2.27(1.61-3.2) | 2.82E-06 |
| rs12454584 | 18p11.31 | 5671544 | - | A | G | 0.07 | 3962 | 0.97 | 0 | 2.27  (1.61-3.2) | 2.82E-06 |
| rs11081208 | 18p11.31 | 5669555 | - | G | A | 0.07 | 3962 | 0.97 | 0 | 2.27  (1.61-3.2) | 2.82E-06 |
| rs16948594 | 18p11.31 | 5664076 | - | G | A | 0.07 | 3962 | 0.97 | 0 | 2.27  (1.61-3.2) | 2.83E-06 |
| rs72634392 | 18p11.31 | 5674148 | - | C | T | 0.07 | 3960 | 0.97 | 0 | 2.27  (1.61-3.2) | 2.87E-06 |
| rs1941014 | 18p11.31 | 5678582 | - | T | C | 0.07 | 3961 | 0.98 | 0 | 2.27  (1.61-3.2) | 2.96E-06 |
| rs894591 | 11q12.2 | 60132647 | - | C | T | 0.65 | 3961 | 0.74 | 0 | 1.54  (1.28-1.84) | 3.28E-06 |
| rs894592 | 11q12.2 | 60132304 | - | G | T | 0.65 | 3961 | 0.74 | 0 | 1.54  (1.28-1.84) | 3.28E-06 |
| rs7611456 | 3p22.2 | 38813465 | SCN10A | T | C | 0.60 | 3960 | 0.17 | 0.46 | 0.66  (0.55-0.79) | 3.41E-06 |
| rs183685693 | 15q21.2 | 52043708 | LYSMD2 | G | C | 0.07 | 3961 | 0.06 | 0.71 | 2.24  (1.59-3.15) | 3.42E-06 |
| rs7936040 | 11q12.2 | 60135392 | - | T | G | 0.65 | 3961 | 0.72 | 0 | 1.53  (1.28-1.84) | 3.54E-06 |
| rs4939349 | 11q12.2 | 60135880 | - | A | G | 0.65 | 3961 | 0.72 | 0 | 1.53  (1.28-1.84) | 3.56E-06 |
| rs4939348 | 11q12.2 | 60135871 | - | C | A | 0.65 | 3961 | 0.72 | 0 | 1.53  (1.28-1.84) | 3.56E-06 |
| rs4939350 | 11q12.2 | 60136243 | - | G | C | 0.65 | 3961 | 0.72 | 0 | 1.53  (1.28-1.84) | 3.57E-06 |
| rs4290252 | 11q12.2 | 60144781 | MS4A7 | T | C | 0.65 | 3961 | 0.72 | 0 | 1.53  (1.28-1.84) | 3.64E-06 |
| rs1319179 | 11q12.2 | 60144141 | MS4A7 | T | C | 0.65 | 3961 | 0.71 | 0 | 1.53  (1.28-1.84) | 3.80E-06 |
| rs7236693 | 18p11.31 | 5636784 | - | C | T | 0.06 | 3961 | 0.89 | 0 | 2.38  (1.65-3.43) | 3.82E-06 |
| rs6591576 | 11q12.2 | 60143407 | - | G | A | 0.65 | 3962 | 0.72 | 0 | 1.53  (1.28-1.84) | 3.82E-06 |
| rs10750936 | 11q12.2 | 60144180 | MS4A7 | A | G | 0.65 | 3962 | 0.72 | 0 | 1.53  (1.28-1.84) | 3.83E-06 |
| rs1318775 | 11q12.2 | 60144133 | MS4A7 | G | A | 0.65 | 3962 | 0.72 | 0 | 1.53  (1.28-1.84) | 3.83E-06 |
| chr11:60144093:D | 11q12.2 | 60144093 | MS4A7 | T | TAC | 0.65 | 3961 | 0.72 | 0 | 1.53  (1.28-1.84) | 3.83E-06 |
| rs4290253 | 11q12.2 | 60144810 | MS4A7 | A | C | 0.65 | 3961 | 0.72 | 0 | 1.53  (1.28-1.84) | 3.84E-06 |
| rs2233238 | 11q12.2 | 60145441 | MS4A7 | C | G | 0.65 | 3962 | 0.71 | 0 | 1.53  (1.28-1.84) | 3.87E-06 |
| rs1838095 | 11q12.2 | 60147347 | MS4A7 | T | C | 0.65 | 3962 | 0.71 | 0 | 1.53  (1.28-1.83) | 3.89E-06 |
| rs188974667 | 14q23.1 | 58244954 | SLC35F4 | A | T | 0.03 | 3961 | 0.05 | 0.73 | 3.4  (2.02-5.71) | 4.03E-06 |
| rs185711677 | 14q23.1 | 58244953 | SLC35F4 | G | A | 0.03 | 3961 | 0.05 | 0.73 | 3.4  (.02-5.71) | 4.03E-06 |
| rs112572084 | 14q23.1 | 58251271 | SLC35F4 | C | T | 0.03 | 3960 | 0.05 | 0.74 | 3.39  (2.02-5.7) | 4.15E-06 |
| rs10897066 | 11q12.2 | 60216445 | - | C | T | 0.54 | 3962 | 0.34 | 0 | 1.5  (1.26-1.79) | 4.36E-06 |
| rs4149064 | 12p12.1 | 21350861 | SLCO1B1 | G | A | 0.05 | 3962 | 0.35 | 0 | 2.6  (1.73-3.91) | 4.44E-06 |
| rs4149063 | 12p12.1 | 21350790 | SLCO1B1 | T | G | 0.05 | 3962 | 0.35 | 0 | 2.6  (1.73-3.91) | 4.44E-06 |
| rs2233239 | 11q12.2 | 60145822 | MS4A7 | A | G | 0.66 | 3961 | 0.55 | 0 | 1.53  (1.28-1.84) | 4.47E-06 |
| rs9504258 | 6p25.1 | 4810647 | CDYL | G | A | 0.02 | 3961 | 0.23 | 0.32 | 3.72  (2.12-6.53) | 4.54E-06 |
| rs9502241 | 6p25.1 | 4807648 | CDYL | C | A | 0.02 | 3960 | 0.22 | 0.32 | 3.72  (2.12-6.52) | 4.55E-06 |
| rs9502240 | 6p25.1 | 4805898 | CDYL | G | C | 0.02 | 3961 | 0.22 | 0.32 | 3.72  (2.12-6.52) | 4.56E-06 |
| rs76124758 | 6p25.1 | 4800863 | CDYL | C | A | 0.02 | 3961 | 0.22 | 0.33 | 3.71  (2.12-6.5) | 4.60E-06 |
| rs12455357 | 18p11.31 | 5672920 | - | A | G | 0.07 | 3961 | 0.87 | 0 | 2.22  (1.58-3.12) | 4.69E-06 |
| chr6:31428746:I | 6p21.33 | 31428746 | - | CATAG | C | 0.13 | 3960 | 0.27 | 0.18 | 1.85  (1.42-2.41) | 4.71E-06 |
| chr14:58240829:D | 14q23.1 | 58240829 | SLC35F4 | A | AG | 0.03 | 3961 | 0.05 | 0.74 | 3.35  (2-5.62) | 4.84E-06 |
| rs12274840 | 11q12.2 | 60275870 | - | G | A | 0.41 | 3960 | 0.77 | 0 | 1.52  (1.27-1.81) | 4.84E-06 |
| rs140544526 | 6p25.1 | 4860293 | CDYL | T | C | 0.06 | 3961 | 0.24 | 0.28 | 2.36  (1.64-3.42) | 4.85E-06 |
| rs62621247 | 6p21.32 | 32521160 | HLA-DRB6/HLA-DRB4 | G | C | 0.19 | 3960 | 0.94 | 0 | 1.84  (1.42-2.39) | 4.89E-06 |
| rs190803379 | 14q23.1 | 58243180 | SLC35F4 | A | C | 0.03 | 3961 | 0.05 | 0.74 | 3.35  (1.99-5.62) | 4.91E-06 |
| rs187962793 | 16p13.12 | 13780571 | - | A | G | 0.02 | 3962 | 0.67 | 0 | 4.4  (2.33-8.31) | 4.96E-06 |
| rs141461675 | 16p13.12 | 13793741 | - | T | C | 0.02 | 3961 | 0.67 | 0 | 4.4  (2.33-8.32) | 5.06E-06 |
| chr6:32489955 | 6p21.32 | 32489955 | HLA-DRB5 | C | T | 0.14 | 3960 | 0.70 | 0 | 1.97  (1.47-2.63) | 5.15E-06 |
| rs114674095 | 6p21.32 | 32289358 | C6orf10 | A | G | 0.17 | 3961 | 0.42 | 0 | 1.74  (1.37-2.21) | 5.21E-06 |
| rs73194696 | 12q21.3 | 90569445 | - | C | T | 0.15 | 3960 | 0.37 | 0 | 1.89  (1.44-2.49) | 5.31E-06 |
| rs10881078 | 12q13.11 | 47598048 | PCED1B | T | A | 0.03 | 3962 | 0.05 | 0.75 | 3.58  (2.07-6.19) | 5.31E-06 |
| rs72792014 | 10q11.22 | 48339911 | - | A | T | 0.03 | 3961 | 0.64 | 0 | 3.37  (2-5.69) | 5.32E-06 |
| rs7952244 | 11q12.2 | 60132164 | - | A | G | 0.65 | 3962 | 0.93 | 0 | 1.52  (1.27-1.82) | 5.39E-06 |
| rs192807392 | 6p21.32 | 32489959 | HLA-DRB5 | T | G | 0.14 | 3960 | 0.68 | 0 | 1.96  (1.47-2.62) | 5.46E-06 |
| rs141170244 | 6p21.32 | 32287402 | C6orf10 | A | C | 0.16 | 3961 | 0.39 | 0 | 1.77  (1.38-2.26) | 5.58E-06 |
| chr6:4808164:I | 6p25.1 | 4808164 | CDYL | AT | A | 0.03 | 3961 | 0.22 | 0.35 | 3.66  (2.09-6.4) | 5.61E-06 |
| rs186391136 | 6p21.32 | 32489507 | HLA-DRB5 | T | G | 0.16 | 3960 | 0.38 | 0 | 1.9  (1.44-2.51) | 5.69E-06 |
| rs72870343 | 18p11.31 | 5648331 | - | T | C | 0.06 | 3960 | 0.74 | 0 | 2.4  (1.64-3.5) | 5.79E-06 |
| rs116322651 | 6p21.32 | 32704362 | - | T | C | 0.21 | 3960 | 0.81 | 0 | 1.65  (1.33-2.04) | 5.81E-06 |
| rs59157296 | 18p11.31 | 5650514 | - | C | T | 0.06 | 3962 | 0.74 | 0 | 2.4  (1.64-3.5) | 5.90E-06 |
| rs189263542 | 15q21.2 | 51853081 | DMXL2 | C | T | 0.07 | 3960 | 0.20 | 0.38 | 2.27  (1.59-3.23) | 5.98E-06 |
| rs114428080 | 3q29 | 194221622 | LINC00884 | A | G | 0.03 | 3961 | 0.06 | 0.71 | 3.56  (2.05-6.16) | 6.10E-06 |
| rs7228452 | 18p11.31 | 5653299 | - | G | A | 0.06 | 3962 | 0.74 | 0 | 2.4  (1.64-3.5) | 6.11E-06 |
| rs2197236 | 11q12.2 | 60168859 | MS4A14 | C | T | 0.63 | 3962 | 0.81 | 0 | 1.51  (1.26-1.81) | 6.15E-06 |
| rs1373064 | 11q12.2 | 60168200 | MS4A14 | T | C | 0.63 | 3961 | 0.81 | 0 | 1.51  (1.26-1.81) | 6.16E-06 |
| rs76691663 | 17q21.33 | 49897602 | CA10 | A | C | 0.07 | 3960 | 0.78 | 0 | 2.65  (1.73-4.04) | 6.48E-06 |
| rs74452455 | 3q29 | 194228309 | - | T | C | 0.03 | 3960 | 0.06 | 0.71 | 3.5  (2.03-6.03) | 6.92E-06 |
| rs7645715 | 3q29 | 194227103 | - | T | C | 0.03 | 3961 | 0.07 | 0.71 | 3.5  (2.03-6.05) | 7.07E-06 |
| rs188957886 | 6q14.1 | 76955002 | - | T | C | 0.02 | 3960 | 0.79 | 0 | 4.56  (2.35-8.84) | 7.27E-06 |
| rs116434681 | 6p21.32 | 32298484 | C6orf10 | T | G | 0.18 | 3962 | 0.49 | 0 | 1.7  (1.35-2.14) | 7.35E-06 |
| rs191212849 | 20q13.33 | 60896518 | LAMA5 | G | A | 0.27 | 3960 | 0.73 | 0 | 1.67  (1.34-2.09) | 7.35E-06 |
| rs116761947 | 6p21.32 | 32644553 | - | T | C | 0.10 | 3961 | 0.67 | 0 | 2.04  (1.49-2.78) | 7.38E-06 |
| rs186599481 | 15q21.2 | 51730272 | - | G | A | 0.07 | 3961 | 0.22 | 0.35 | 2.25  1.58-3.21) | 7.40E-06 |
| rs145359782 | 6p21.32 | 32302672 | C6orf10 | A | G | 0.17 | 3962 | 0.37 | 0 | 1.72  (1.36-2.19) | 7.45E-06 |
| chr6:32299524:D | 6p21.32 | 32299524 | C6orf10 | C | CA | 0.17 | 3961 | 0.36 | 0 | 1.72  (1.36-2.19) | 7.63E-06 |
| rs115461021 | 6p21.32 | 32310119 | C6orf10 | G | T | 0.17 | 3962 | 0.36 | 0 | 1.72  (1.36-2.19) | 7.66E-06 |
| rs115743005 | 6p21.32 | 32307532 | C6orf10 | A | G | 0.17 | 3962 | 0.36 | 0 | 1.72  (1.36-2.19) | 7.66E-06 |
| rs116397353 | 6p21.32 | 32294549 | C6orf10 | T | A | 0.17 | 3962 | 0.36 | 0 | 1.72  (1.36-2.19) | 7.66E-06 |
| rs140714356 | 6p21.32 | 32293941 | C6orf10 | C | G | 0.17 | 3962 | 0.36 | 0 | 1.72  (1.36-2.19) | 7.66E-06 |
| rs114944682 | 6p21.32 | 32287754 | C6orf10 | C | T | 0.17 | 3962 | 0.36 | 0 | 1.72  (1.36-2.19) | 7.66E-06 |
| rs115256525 | 6p21.32 | 32266795 | C6orf10 | A | G | 0.17 | 3962 | 0.36 | 0 | 1.72  (1.36-2.19) | 7.66E-06 |
| rs114136185 | 6p21.32 | 32264179 | C6orf10 | T | C | 0.17 | 3962 | 0.36 | 0 | 1.72  (1.36-2.19) | 7.66E-06 |
| rs114616323 | 6p21.32 | 32263458 | C6orf10 | G | T | 0.17 | 3962 | 0.36 | 0 | 1.72  (1.36-2.19) | 7.66E-06 |
| rs116558425 | 6p21.32 | 32255507 | - | G | A | 0.17 | 3962 | 0.36 | 0 | 1.72  (1.36-2.19) | 7.66E-06 |
| rs115172733 | 6p21.32 | 32253559 | - | C | T | 0.17 | 3962 | 0.36 | 0 | 1.72  (1.36-2.19) | 7.66E-06 |
| rs145499705 | 6p21.32 | 32237260 | - | G | A | 0.17 | 3962 | 0.36 | 0 | 1.72  (1.36-2.19) | 7.66E-06 |
| rs115912164 | 6p21.32 | 32234993 | - | G | A | 0.17 | 3962 | 0.36 | 0 | 1.72  (1.36-2.19) | 7.66E-06 |
| rs114141321 | 6p21.32 | 32336161 | C6orf10 | A | G | 0.17 | 3962 | 0.36 | 0 | 1.72  (1.36-2.19) | 7.67E-06 |
| rs116105456 | 6p21.32 | 32252507 | - | G | A | 0.17 | 3962 | 0.36 | 0 | 1.72  (1.36-2.19) | 7.67E-06 |
| rs111703821 | 6p21.32 | 32223109 | - | G | A | 0.17 | 3960 | 0.36 | 0 | 1.72  (1.36-2.19) | 7.67E-06 |
| rs4822899 | 22q12.1 | 27919967 | - | C | A | 0.47 | 3962 | 0.15 | 0.52 | 1.48  (1.25-1.76) | 7.68E-06 |
| rs7242770 | 18p11.31 | 5647875 | - | A | G | 0.06 | 3962 | 0.83 | 0 | 2.35  (1.62-3.43) | 7.71E-06 |
| rs72870358 | 18p11.31 | 5655667 | - | C | G | 0.06 | 3961 | 0.73 | 0 | 2.38  1.63-3.48) | 7.79E-06 |
| chr13:46887522:D | 13q14.13 | 46887522 | - | A | AAT | 0.03 | 3961 | 0.41 | 0 | 4.02  (2.19-7.41) | 7.86E-06 |
| rs186127431 | 12q13.11 | 47876807 | - | C | T | 0.02 | 3961 | 0.09 | 0.64 | 3.99  (2.17-7.31) | 7.94E-06 |
| rs115250958 | 6p21.32 | 32509842 | HLA-DRB5 | A | C | 0.08 | 3961 | 0.81 | 0 | 2.37  (1.62-3.46) | 8.11E-06 |
| rs4938941 | 11q12.2 | 60173360 | MS4A14 | G | A | 0.63 | 3961 | 0.75 | 0 | 1.5  (1.26-1.8) | 8.62E-06 |
| rs7946122 | 11q12.2 | 60172770 | MS4A14 | A | G | 0.63 | 3961 | 0.75 | 0 | 1.5  (1.26-1.8) | 8.69E-06 |
| rs74194578 | 6p21.32 | 32517590 | HLA-DRB5 | T | C | 0.29 | 3960 | 0.34 | 0 | 1.65  (1.32-2.05) | 8.84E-06 |
| rs10897057 | 11q12.2 | 60172273 | MS4A14 | T | A | 0.63 | 3961 | 0.76 | 0 | 1.5  (1.26-1.8) | 8.84E-06 |
| rs10750937 | 11q12.2 | 60172087 | MS4A14 | T | C | 0.63 | 3962 | 0.76 | 0 | 1.5  (1.26-1.8) | 8.85E-06 |
| rs116379234 | 6p21.32 | 32295878 | C6orf10 | A | G | 0.18 | 3961 | 0.44 | 0 | 1.69  (1.34-2.13) | 8.91E-06 |
| rs79744005 | 6p25.1 | 4884129 | CDYL | G | A | 0.02 | 3961 | 0.27 | 0.18 | 3.53  (2.02-6.16) | 9.01E-06 |
| rs79724978 | 3q29 | 194224763 | - | T | C | 0.03 | 3962 | 0.08 | 0.68 | 3.44  (2-5.94) | 9.01E-06 |
| rs114725367 | 6p21.32 | 32316312 | C6orf10 | C | T | 0.18 | 3960 | 0.43 | 0 | 1.69  (1.34-2.13) | 9.24E-06 |
| rs186243288 | 9q21.31 | 82083169 | - | G | A | 0.05 | 3961 | 0.63 | 0 | 2.82  (1.78-4.45) | 9.34E-06 |
| rs113018536 | 9p24.1 | 4695330 | CDC37L1 | T | C | 0.06 | 3961 | 0.33 | 0 | 2.4  (1.63-3.54) | 9.41E-06 |
| rs1003878 | 6p21.32 | 32299822 | C6orf10 | A | G | 0.18 | 3962 | 0.44 | 0 | 1.69  (1.34-2.12) | 9.43E-06 |
| rs4340070 | 11q12.2 | 60203209 | MS4A5 | T | C | 0.55 | 3960 | 0.84 | 0 | 1.5  (1.25-1.8) | 9.64E-06 |
| rs75292430 | 11q12.2 | 60277982 | - | A | G | 0.44 | 3960 | 0.83 | 0 | 1.51  (1.26-1.81) | 9.69E-06 |
| rs116282195 | 6p21.32 | 32305658 | C6orf10 | A | C | 0.18 | 3962 | 0.45 | 0 | 1.69  (1.34-2.13) | 9.83E-06 |
| rs115132660 | 6p21.32 | 32301910 | C6orf10 | A | G | 0.18 | 3962 | 0.45 | 0 | 1.69  (1.34-2.13) | 9.83E-06 |
| rs114749621 | 6p21.32 | 32298814 | C6orf10 | A | C | 0.18 | 3962 | 0.45 | 0 | 1.69  (1.34-2.13) | 9.83E-06 |
| rs12359953 | 10p11.22 | 33358070 | - | T | C | 0.07 | 3962 | 0.45 | 0 | 2.07  (1.5-2.87) | 9.93E-06 |

^$^Chromosomal positions are based on NCBI build 37. Abbreviations: SNP, single nucleotide polymorphism; Chr, chromosome; N, number of subjects; Phet, Cochran’s heterogeneity statistic’s p-value; I^2^, heterogeneity index (I^2^<0.75); OR, odds ratio; CI; confidence intervals, NA, not applicable.
