# Supplementary material for: Genome-Wide Meta-Analysis of Sciatica in Finnish Population
Source: PLoS One. 2016 Oct 20;11(10):e0163877. doi: 10.1371/journal.pone.0163877 (PMC5072673; doi:10.1371/journal.pone.0163877)
Supplement: S3 Table — (DOCX) [file pone.0163877.s010.docx]

##

## Supplementary Table S3. Variants associated with sciatica in the meta-analysis (p < 1x10^-6^) with effect allele frequencies, imputation quality and p-values in two discovery GWASes.

| **SNP** | **Chr** | **Position^$^** | **Gene** | **N** | **Effect allele** | **Other allele** | **YFS** | **H2000** | **YFS** | **H2000** | **YFS** | **H2000** | **Meta-analysis** | | |
| --- | --- | --- | --- | --- | --- | --- | --- | --- | --- | --- | --- | --- | --- | --- | --- |
|  |  |  |  |  |  |  | **Effect allele freq.** | | **Imputation quality^#^** | | **P value*** | | **P_het_** | ***I^2^*** | **P value** |
| chr9:14344410:I | 9p22.3 | 14344410 | NFIB | 3960 | AG | A | 0.08 | 0.07 | 0.78 | 0.77 | 4.09E-06 | 3.54E-4 | 0.82 | 0 | 1.30E-08 |
| rs145901849 | 15q21.2 | 52640539 | MYO5A | 3961 | T | C | 0.06 | 0.06 | 0.91 | 0.93 | 3.83E-08 | 0.028 | 0.11 | 0.61 | 1.34E-08 |
| rs80035109 | 15q21.2 | 52665890 | MYO5A | 3962 | C | T | 0.07 | 0.07 | 0.97 | 0.97 | 3.73E-07 | 9.96E-3 | 0.32 | 0 | 2.32E-08 |
| rs190200374 | 15q21.2 | 52811959 | MYO5A | 3962 | T | G | 0.06 | 0.06 | 0.84 | 0.87 | 6.72E-08 | 0.047 | 0.10 | 0.63 | 3.85E-08 |
| rs117458827 | 15q21.2 | 52600066 | MYO5A | 3960 | A | G | 0.07 | 0.07 | 0.99 | 0.99 | 8.19E-07 | 9.98E-3 | 0.35 | 0 | 4.78E-08 |
| rs149430802 | 15q21.3 | 52948838 | FAM214A | 3961 | T | C | 0.07 | 0.07 | 0.95 | 0.96 | 3.87E-07 | 0.029 | 0.20 | 0.38 | 8.48E-08 |
| rs117288416 | 15q21.3 | 52957840 | FAM214A | 3961 | T | C | 0.07 | 0.07 | 0.95 | 0.96 | 3.87E-07 | 0.029 | 0.20 | 0.38 | 8.49E-08 |
| chr15:52604566:I | 15q21.2 | 52604566 | MYO5A | 3960 | CT | C | 0.07 | 0.07 | 0.92 | 0.92 | 1.51E-06 | 0.011 | 0.39 | 0 | 8.58E-08 |
| rs143229532 | 15q21.3 | 52928933 | FAM214A | 3961 | C | T | 0.07 | 0.07 | 0.95 | 0.96 | 3.95E-07 | 0.029 | 0.20 | 0.39 | 8.86E-08 |
| rs117930495 | 15q21.2 | 52743583 | MYO5A | 3961 | C | T | 0.07 | 0.07 | 0.97 | 0.98 | 6.28E-07 | 0.027 | 0.21 | 0.35 | 1.24E-07 |
| rs80026449 | 11q12.2 | 60317643 | - | 3962 | A | G | 0.03 | 0.03 | 0.80 | 0.78 | 8.15E-07 | 0.019 | 0.19 | 0.41 | 1.62E-07 |
| rs183165962 | 15q21.2 | 52514666 | MYO5C | 3962 | A | G | 0.07 | 0.06 | 0.90 | 0.93 | 5.62E-07 | 0.043 | 0.15 | 0.51 | 2.16E-07 |
| chr15:52852285:I | 15q21.2 | 52852285 | ARPP19 | 3961 | TA | T | 0.07 | 0.07 | 0.94 | 0.95 | 7.94E-07 | 0.041 | 0.19 | 0.43 | 2.48E-07 |
| rs190606317 | 6p21.32 | 32508053 | - | 3960 | A | G | 0.17 | 0.14 | 0.70 | 0.71 | 7.24E-4 | 9.56E-06 | 0.11 | 0.61 | 2.74E-07 |
| rs73937196 | 18p11.31 | 5636910 | - | 3962 | T | C | 0.0 | 0.05 | 0.94 | 0.93 | 7.60E-05 | 5.95E-4 | 0.77 | 0 | 2.95E-07 |
| rs115488695 | 6p21.32 | 32490036 | HLA-DRB5 | 3960 | T | C | 0.21 | 0.15 | 0.79 | 0.76 | 1.69E-05 | 3.95E-3 | 0.85 | 0 | 3.58E-07 |
| rs58509608 | 18p11.31 | 5640484 | - | 3961 | C | T | 0.05 | 0.05 | 0.95 | 0.94 | 1.2E-4 | 5.84E-4 | 0.71 | 0 | 4.52E-07 |
| rs186767095 | 15q21.2 | 52388742 | - | 3960 | A | T | 0.06 | 0.06 | 0.83 | 0.88 | 5.01E-07 | 0.099 | 0.08 | 0.68 | 6.53E-07 |
| rs62100562 | 18q22.3 | 71537484 | - | 3961 | T | G | 0.02 | 0.03 | 0.82 | 0.84 | 3.39E-3 | 1.85E-05 | 0.21 | 0.35 | 6.61E-07 |
| rs115949512 | 6p21.33 | 31430721 | HCP5 | 3961 | G | A | 0.13 | 0.14 | 0.99 | 0.99 | 1.29E-05 | 8.79E-3 | 0.29 | 0.09 | 7.30E-07 |
| rs117146116 | 9p21.1 | 28313102 | LINGO2 | 3961 | C | T | 0.03 | 0.03 | 0.84 | 0.84 | 2.51E-4 | 3.86E-4 | 0.58 | 0 | 7.50E-07 |
| rs3094014 | 6p21.33 | 31433558 | HCP5 | 3962 | A | G | 0.13 | 0.14 | 1 | 1 | 7.94E-06 | 0.014 | 0.22 | 0.33 | 8.03E-07 |
| rs2241921 | 11q12.2 | 60164302 | MS4A14 | 3962 | T | C | 0.36 | 0.37 | 1 | 1 | 7.41E-4 | 2.79E-4 | 0.52 | 0 | 8.93E-07 |
| rs6591578 | 11q12.2 | 60158649 | MS4A7 | 3962 | A | G | 0.36 | 0.36 | 0.99 | 0.99 | 6.23E-4 | 2.99E-4 | 0.56 | 0 | 9.19E-07 |
| rs114615271 | 6p21.33 | 31434198 | - | 3960 | C | T | 0.13 | 0.13 | 0.98 | 0.99 | 8.85E-06 | 0.014 | 0.22 | 0.33 | 9.51E-07 |
| rs10145254 | 14q13.1 | 34534344 | - | 3960 | T | C | 0.03 | 0.02 | 0.82 | 0.83 | 1.34E-05 | 0.013 | 0.41 | 0 | 9.62E-07 |
| rs115688765 | 6p21.33 | 31433831 | HCP5 | 3962 | G | A | 0.13 | 0.14 | 0.99 | 0.99 | 9.16E-06 | 0.014 | 0.23 | 0.32 | 9.79E-07 |
| rs10792269 | 11q12.2 | 60117126 | - | 3962 | A | G | 0.35 | 0.36 | 1 | 1 | 3.54E-4 | 7.73E-4 | 0.77 | 0 | 9.91E-07 |
| rs77310140 | 4p15.1 | 35532585 | - | 3962 | G | A | 0.03 | 0.04 | 0.73 | 0.78 | 6.85E-05 | 0.003 | 0.61 | 0 | 9.97E-07 |

^$^Chromosomal positions are based on NCBI build 37; ^#^Imputation quality score from IMPUTE; *Additive model, adjusted for seven first principal components, age and gender. Abbreviations: SNP, single nucleotide polymorphism; Chr, chromosome; N, number of subjects; YFS, Young Finns Study; H2000, Health 2000 Study; Phet, Cochran’s heterogeneity statistic’s p-value; I^2^, heterogeneity index (I^2^<0.75).
