# Supplementary material for: Genome-Wide Meta-Analysis of Sciatica in Finnish Population
Source: PLoS One. 2016 Oct 20;11(10):e0163877. doi: 10.1371/journal.pone.0163877 (PMC5072673; doi:10.1371/journal.pone.0163877)
Supplement: S4 Table — (DOCX) [file pone.0163877.s011.docx]

## Supplementary Table S4. LD estimates from the YFS (upper diagonal in green) and H2000 (lower diagonal in yellow) for SNPs with p<5x10^-8^ within the locus 15q21.2.

|  | **r^2^** | | | |
| --- | --- | --- | --- | --- |
| **SNP** | **rs145901849** | **rs80035109** | **rs190200374** | **rs117458827** |
| **rs145901849** | 1 | 0.88 | 0.89 | 0.87 |
| **rs80035109** | 0.85 | 1 | 0.83 | 0.99 |
| **rs190200374** | 0.89 | 0.83 | 1 | 0.81 |
| **rs117458827** | 0.85 | 0.99 | 0.89 | 1 |

Abbreviations: YFS, Young Finns Study; H2000, Health 2000 Study; SNP, single nucleotide polymorphism; r^2^, R-squared.
