# Supplementary material for: Genome-Wide Meta-Analysis of Sciatica in Finnish Population
Source: PLoS One. 2016 Oct 20;11(10):e0163877. doi: 10.1371/journal.pone.0163877 (PMC5072673; doi:10.1371/journal.pone.0163877)
Supplement: S5 Table — (DOCX) [file pone.0163877.s012.docx]

**Table S5. Minor allele frequencies for the five most promising variants in the Finnish and other European populations.**

| **SNP** | **Chr** | **Gene** | **EA/OA** | **MAF (%)** | | | | | | | |
| --- | --- | --- | --- | --- | --- | --- | --- | --- | --- | --- | --- |
|  |  |  |  | **YFS*** | **H2000*** | **1000 Genomes Project** | | | | | |
|  |  |  |  |  |  | **FIN** | **CEU** | **GBR** | **IBS** | **TSI** | **EUR** |
| chr9:14344410:I rs71321981 | 9p22.3 | *NFIB* | G/- | 8 | 7 | 7 | 8 | 6 | 8 | 9 | 8 |
| rs145901849 | 15q21.2 | *MYO5A* | T/C | 6 | 6 | 1 | 0 | 0 | 0 | 0 | 0 |
| rs80035109 | 15q21.2 | *MYO5A* | C/T | 7 | 7 | 5 | 2 | 2 | 1 | 0 | 2 |
| rs190200374 | 15q21.2 | *MYO5A* | T/G | 6 | 6 | 5 | 2 | 1 | 0 | 0 | 1 |
| rs117458827 | 15q21.2 | *MYO5A* | A/G | 7 | 7 | 5 | 2 | 2 | 1 | 0 | 2 |

Abbreviations: Chr, chromosomal locus; EA, effect allele/minor allele; OA, other allele; MAF, minor allele frequency; YFS, Young Finns Study discovery cohort; H2000, Health 2000 Study discovery cohort; FIN, Finnish in Finland; CEU, Utah residents with Northern and Western European ancestry; GBR; British in England and Scotland; IBS, Iberian populations in Spain; TSI; Toscani in Italy, EUR, European (1000 Genomes Project, see URLs).
