# Supplementary material for: Genome-Wide Meta-Analysis of Sciatica in Finnish Population
Source: PLoS One. 2016 Oct 20;11(10):e0163877. doi: 10.1371/journal.pone.0163877 (PMC5072673; doi:10.1371/journal.pone.0163877)
Supplement: S6 Table — (DOCX) [file pone.0163877.s013.docx]

## Supplementary Table S6. LD estimates from the YFS (upper diagonal in green) and H2000 (lower diagonal in yellow) for the SNPs in the HLA region within the locus 6p21.32.

|  | **r^2^** | | | | | | | | | |
| --- | --- | --- | --- | --- | --- | --- | --- | --- | --- | --- |
| **SNP** | **rs190606317^@^** | **rs115488695^@^** | **rs115949512^@^** | **rs3094014^@^** | **rs114615271^@^** | **rs115688765^@^** | **rs7775228^#^** | **rs10947262^#^** | **rs2076311^¤^** | **rs1799907^*^** |
| **rs190606317^@^** | 1 | 0.31 | 0.2 | 0.91 | 0.23 | 0.2 | 0.01 | 0.00 | 0.05 | 0.05 |
| **rs115488695^@^** | 0.36 | 1 | 0.06 | 0.07 | 0.08 | 0.07 | 0.02 | 0.11 | 0.02 | 0.02 |
| **rs115949512^@^** | 0.17 | 0.06 | 1 | 0.98 | 0.98 | 0.98 | 0.02 | 0.01 | 0.04 | 0.04 |
| **rs3094014^@^** | 0.17 | 0.06 | 0.97 | 1 | 0.99 | 0.99 | 0.02 | 0.01 | 0.05 | 0.05 |
| **rs114615271^@^** | 0.17 | 0.06 | 0.97 | 1 | 1 | 1 | 0.02 | 0.02 | 0.05 | 0.05 |
| **rs115688765^@^** | 0.17 | 0.06 | 0.97 | 1 | 1 | 1 | 0.02 | 0.01 | 0.04 | 0.04 |
| **rs7775228^#^** | 0.02 | 0.01 | 0.03 | 0.02 | 0.02 | 0.2 | 1 | 0.24 | 0.007 | 0.007 |
| **rs10947262^#^** | 0.1 | 0.09 | 0.02 | 0.02 | 0.02 | 0.02 | 0.26 | 1 | 0.00 | 0.00 |
| **rs2076311^¤^** | 0.01 | 0.02 | 0.04 | 0.03 | 0.03 | 0.03 | 0.01 | 0.00 | 1 | 0.99 |
| **rs1799907^*^** | 0.01 | 0.02 | 0.03 | 0.03 | 0.03 | 0.03 | 0.01 | 0.00 | 0.99 | 1 |

^@^Associated with sciatica with p<1x10^-6^ in the present study; ^#^previously associated with osteoarthritis ([1](#_ENREF_1)); ^¤^previously associated with magnetic resonance-determined disc signal intensity ([2](#_ENREF_2)); *previously associated with degenerative lumbar spinal stenosis with radicular pain ([3](#_ENREF_3)). Abbreviations: YFS, Young Finns Study; H2000, Health 2000 study; SNP, single nucleotide polymorphism; r^2^, R-squared.
