# Supplementary material for: Genome-Wide Meta-Analysis of Sciatica in Finnish Population
Source: PLoS One. 2016 Oct 20;11(10):e0163877. doi: 10.1371/journal.pone.0163877 (PMC5072673; doi:10.1371/journal.pone.0163877)
Supplement: S7 Table — (DOCX) [file pone.0163877.s014.docx]

## Supplementary Table S7. The most promising variants in the GWAS meta-analysis of sciatica (p<1x10^-6^) as tested for replication in a Finnish population-based cohort (FINRISK).

| **SNP** | **Chr** | **Position** | | **Gene** | **EA/OA** | **EAF** | | | **Imputation quality** | **OR**  **(95% CI)** | **P value*** |
| --- | --- | --- | --- | --- | --- | --- | --- | --- | --- | --- | --- |
|  |  |  |  |  |  | **all** | **case** | **cntrl** |  |  |  |
| chr9:14344410:I | 9p22.3 | 14344410 | NFIB | | AG/G | 0.07 | 0.08 | 0.07 | 0.59 | 1.17  (0.97-1.40) | 0.04 |
| rs190606317 | 6p21.32^#^ | 32508053 | - | | A/G | 0.18 | 0.16 | 0.18 | 0.74 | 0.85  (0.74-0.97) | 0.006 |
| rs62100562 | 18q22.3 | 71537484 | - | | T/G | 0.03 | 0.02 | 0.03 | 0.85 | 0.73  (0.52-1.01) | 0.03 |
| rs117146116 | 9p21.1 | 28313102 | LINGO2 | | C/T | 0.03 | 0.02 | 0.03 | 0.89 | 0.79  (0.56- 1.11) | 0.13 |
| rs145901849 | 15q21.2 | 52640539 | MYO5A | | T/C | 0.003 | 0.004 | 0.003 | 0.53 | 1.42  (0.66-3.04) | 0.22 |
| rs143229532 | 15q21.3 | 52928933 | FAM214A | | C/T | 0.07 | 0.08 | 0.07 | 0.92 | 1.12  (0.92-1.35) | 0.25 |
| rs149430802 | 15q21.3 | 52948838 | FAM214A | | T/C | 0.07 | 0.08 | 0.07 | 0.93 | 1.12  (0.92-1.35) | 0.25 |
| rs117288416 | 15q21.3 | 52957840 | FAM214A | | T/C | 0.07 | 0.08 | 0.07 | 0.93 | 1.12  (0.92-1.35) | 0.25 |
| rs80035109 | 15q21.2 | 52665890 | MYO5A | | C/T | 0.07 | 0.07 | 0.07 | 0.83 | 1.11  (0.91-1.35) | 0.25 |
| rs114615271 | 6p21.33^#^ | 31434198 | NA | | C/T | 0.13 | 0.12 | 0.13 | 0.97 | 0.91851  (0.79-1.07) | 0.28 |
| rs115688765 | 6p21.33^#^ | 31433831 | HCP5 | | G/A | 0.13 | 0.12 | 0.13 | 0.98 | 0.92  (0.79-1.08) | 0.28 |
| rs3094014 | 6p21.33^#^ | 31433558 | HCP5 | | A/G | 0.13 | 0.12 | 0.13 | 0.98 | 0.92  (0.79-1.08) | 0.29 |
| rs115949512 | 6p21.33^#^ | 31430721 | HCP5 | | G/A | 0.13 | 0.12 | 0.13 | 0.99 | 0.92  (0.79-1.08) | 0.29 |
| rs117930495 | 15q21.2 | 52743583 | MYO5A | | C/T | 0.07 | 0.08 | 0.07 | 0.91 | 1.10  (0.91-1.33) | 0.30 |
| rs190200374 | 15q21.2 | 52811959 | MYO5A | | T/G | 0.07 | 0.07 | 0.07 | 0.93 | 1.10  (0.90-1.34) | 0.33 |
| chr15:52852285:I | 15q21.2 | 52852285 | ARPP19 | | TA/T | 0.07 | 0.07 | 0.07 | 0.93 | 1.09  (0.90-1.33) | 0.37 |
| rs73937196 | 18p11.31 | 5636910 | - | | T/C | 0.06 | 0.05 | 0.06 | 0.96 | 0.91  (0.72-1.14) | 0.39 |
| rs58509608 | 18p11.31 | 5640484 | - | | C/T | 0.06 | 0.05 | 0.05 | 0.97 | 0.91  (0.72-1.14) | 0.39 |
| chr15:52604566:I | 15q21.2 | 52604566 | MYO5A | | CT/C | 0.06 | 0.07 | 0.06 | 0.92 | 1.08  (0.88-1.32) | 0.44 |
| rs183165962 | 15q21.2 | 52514666 | MYO5C | | A/G | 0.06 | 0.07 | 0.06 | 0.92 | 1.08  (0.88-1.32) | 0.44 |
| rs10792269 | 11q12.2 | 60117126 | NA | | A/G | 0.36 | 0.37 | 0.36 | 0.99 | 0.97  (0.87-1.07) | 0.50 |
| rs117458827 | 15q21.2 | 52600066 | MYO5A | | A/G | 0.07 | 0.08 | 0.07 | 0.92 | 1.06  (0.88-1.29) | 0.51 |
| rs186767095 | 15q21.2 | 52388742 | NA | | A/T | 0.03 | 0.03 | 0.03 | 0.66 | 1.07  (0.81-1.42) | 0.54 |
| rs115488695 | 6p21.32^#^ | 32490036 | HLA-DRB5 | | T/C | 0.06 | 0.06 | 0.06 | 0.65 | 1.05  (0.86-1.30) | 0.55 |
| rs186280351 | 15q21.2 | 52024775 | LYSMD2 | | T/G | 0.003 | 0.004 | 0.003 | 0.75 | 1.15  (0.50-2.61) | 0.71 |
| rs10145254 | 14q13.1 | 34534344 | - | | T/C | 0.03 | 0.03 | 0.03 | 0.69 | 1.03  (0.77-1.37) | 0.81 |
| rs6591578 | 11q12.2 | 60158649 | MS4A7 | | A/G | 0.37 | 0.38 | 0.37 | 0.99 | 0.99  (0.89-1.10) | 0.85 |
| rs77310140 | 4p15.1 | 35532585 | - | | G/A | 0.03 | 0.03 | 0.03 | 0.80 | 1.03  (0.75-1.41) | 0.86 |
| rs2241921 | 11q12.2 | 60164302 | MS4A14 | | T/C | 0.37 | 0.38 | 0.37 | 0.99 | 0.99  (0.89- 1.10) | 0.88 |
| rs80026449 | 11q12.2 | 60317643 | - | | A/G | 0.03 | 0.03 | 0.03 | 0.89 | 1.00  (0.73-1.37) | 0.97 |

*SNPTEST v2: frequentist association test for additive genetic model, ^#^HLA gene region. Abbreviations: SNP, single nucleotide polymorphism; Chr, chromosomal locus; EA, effect allele; OA; other allele; EAF, effect allele frequency; OR (95% CI), odds ratio (95% confidence interval).
