# Supplementary material for: Genome-Wide Meta-Analysis of Sciatica in Finnish Population
Source: PLoS One. 2016 Oct 20;11(10):e0163877. doi: 10.1371/journal.pone.0163877 (PMC5072673; doi:10.1371/journal.pone.0163877)
Supplement: S8 Table — (DOCX) [file pone.0163877.s015.docx]

**Table S8. Genotype validations.** Three variants showing genome-wide significant association in the GWAS meta-analysis were subsequently genotyped in individuals of the Young Finns Study and Health 2000 discovery cohorts.

| **SNP** | **Chr** | **Gene** | **EA/OA** | **Study group** | **Young Finns Study** | | | | | **Health 2000** | | | | |
| --- | --- | --- | --- | --- | --- | --- | --- | --- | --- | --- | --- | --- | --- | --- |
|  |  |  |  |  | **Number genotyped (Concordance)^§^** | **Imputation quality^#^ in GWAS** | **EAF** | **OR**  **(95% CI)** | **P-value*** | **Number genotyped**  **(Concordance)^§^** | **Imputation quality^#^ in GWAS** | **EAF** | **OR**  **(95% CI)** | **P-value*** |
| **chr9:14344410:I;**  **rs71321981** | 9p22.3 | *NFIB* | G/- | Sciatica | 92 | - | 0.10 | NP | NP | 89 | - | 0.13 | NP | NP |
|  |  |  |  | Control | 92 | - | 0.10 | Ref | Ref | 95 | - | 0.09 | Ref | Ref |
|  |  |  |  | Total | 184  (88.2%) | 0.78 | 0.10 | - | - | 184  (87.3%) | 0.77 | 0.11 | - | - |
| **rs190200374** | 15q21.2 | *MYO5A* | T/G | Sciatica | 152 | - | 0.13 | 2.03  (1.66-2.4) | 2.21x10^-4^ | 82 | - | 0.12 | 1.95  (1.44-2.46) | 9.70x10^-3^ |
|  |  |  |  | Control | 1534 | - | 0.06 | Ref | Ref | 1323 | - | 0.07 | Ref | Ref |
|  |  |  |  | Total | 1686  (96.7%) | 0.84 | 0.07 | - | - | 1405  (96.2%) | 0.87 | 0.07 | - | - |
| **rs80035109** | 15q21.2 | *MYO5A* | C/T | Sciatica | 154 | - | 0.13 | 2.18  (1.81-2.55) | 4.39x10^-5^ | 99 | - | 0.11 | 1.88  (1.39-2.37) | 1.07x10^-2^ |
|  |  |  |  | Control | 1488 | - | 0.06 | Ref | Ref | 1293 | - | 0.07 | Ref | Ref |
|  |  |  |  | Total | 1642  (99.6%) | 0.97 | 0.07 | - | - | 1392  (98.3%) | 0.97 | 0.07 | - | - |

§Number of genotyped individuals who were part of the respective discovery GWAS, with concordances (%) between genotyped and imputed SNPs using threshold 0.7 for converting probabilistic genotypes to hard calls. #Imputation quality score from IMPUTE; *Logistic regression model, results adjusted by sex, age, 7 principal components. Abbreviations: Chr, chromosomal locus; - , not applicable; EA, effect allele; OA, other allele; EAF, effect allele frequency; OR (95% CI), odds ratio (95% confidence interval); Ref, reference; NP, no power to test association for this variant, because individuals were selected for concordance calculations by genotype (emphasizing EA heterozygotes and homozygotes).
