# Supplementary material for: Genome-Wide Meta-Analysis of Sciatica in Finnish Population
Source: PLoS One. 2016 Oct 20;11(10):e0163877. doi: 10.1371/journal.pone.0163877 (PMC5072673; doi:10.1371/journal.pone.0163877)
Supplement: S1 Text — (DOCX) [file pone.0163877.s016.docx]

## Supporting information: S1 Text

## Study populations and phenotypes

The two Finnish population cohorts (The Young Finns Study and the Health 2000 Study) investigated and subsequently included in the meta-analysis, as well as the population study used for replication purposes (the FINRISK Study), are representative cohorts of the Finnish population. Study populations are described in detail below.

**The Young Finns Study**

The Young Finns Study is a population based prospective cohort study ([see](http://www.ncbi/nih/gov) URLs). It is an on-going follow-up study of atherosclerosis risk factors and its precursors from childhood to adulthood, conducted among those living in or at about 100 km distance from five university cities in Finland (Helsinki, Kuopio, Oulu, Tampere and Turku). A total of 3600 subjects participated in the study at initiation in 1980 (n=3600), of whom 2800 (78%) participated in the follow-up in 1986, 2600 (72%) in 2001 and 2204 (61%) in 2007. In 2007 the participants were 30-45 years old. The follow-up on-site examinations included self-administered questionnaires in 2001 and 2007 with questions on low back pain and disorders, including information on self-reported physician-diagnosed sciatica (“Do you currently have or have you had a long-term disease diagnosed by a physician, such as sciatica?”). The cohort profile has been described in detail previously [4].

**The Health 2000 Study**

The Health 2000 Study is a national health examination survey ([see](http://www.ncbi/nih/gov) URLs). A nationally representative population sample of individuals aged 30 or over and living in mainland Finland (in or nearby Helsinki, Kuopio, Oulu, Tampere and Turku) between the fall of 2000 and the spring of 2001 was formed with a two-stage cluster sampling method [5]. Of the sample of 8028 persons, 6354 (80%) attended a comprehensive health examination. Specially trained field physicians worked according to detailed written instructions with preset diagnostic criteria. In the presence of chronic (>3 months) low back pain radiating down to the leg, and either a positive finding suggesting nerve root compression or a history of lumbar disc herniation that had previously been confirmed by a radiographic examination or required surgery [[5](#_ENREF_5)]. The study has been described in detail elsewhere [[6](#_ENREF_6)]. A subpopulation of the H2000 Study population has been included in a genome-wide scan used in the GWAS; those comprise individuals fulfilling a set of metabolic syndrome criteria and matched controls [[7](#_ENREF_7)].

**The FINRISK study**

The FINRISK Study consists of cross-sectional population surveys carried out at five-year intervals to study risk factors of chronic non-communicable diseases in Finland [[8](#_ENREF_8)]. Since 1972, a random sample of about 8000 individuals has been drawn every five years from the national population register for different parts of Finland (Eastern, Southwestern, and Northern Finland, and the cities of Helsinki and Vantaa as the metropolitan area). The FINRISK random samples were drawn stratified by sex and 10-year age group from the population aged 25-74. The current study population comprised cohorts from the surveys conducted in 1992, 1997, 2002, and 2007. Participation rate was for men 57%-76% and for women 69%-85% in the four surveys included in the replication cohort, resulting in an approximately equal representation of men and women (47% men, 53% women) [[8](#_ENREF_8)] (FINRISK; see URLs). Based on linkage of the FINRISK Study populations to the Finnish Hospital Discharge Register (currently the Finnish Care Register for Health Care) (see URL), personal identification code-based individual diagnoses (WHO ICD-codes) at discharge were available. For the replication study, ICD-codes relevant for sciatica or sciatic syndrome selected *a priori* by two physicians with expertise in musculoskeletal diseases (EVJ and MH) (ICD8 353, 728.8; ICD9 724.3, 722.1, 722.10, 722.5, 722.52, 355.0; ICD10 M54.3, M51.1, M54.1, M54.4) were used to identify cases (amounting to 776 sciatica cases and 18,489 controls) (see URLs; Table 1).

This study was carried out in accordance with the recommendations of the Declaration of Helsinki. All participants of studies have given an informed consent. Studies were approved by the local research ethic committees: Ethics Committee of the National Public Health Institute for H2000, Ethics Committee of the Hospital District of Southwest Finland for YFS and Ethics Committee of Helsinki and Uusimaa Hospital for FINRISK.

References cited in Supporting information are given in S2 Text.
