# Supplementary material for: Genome-Wide Meta-Analysis of Sciatica in Finnish Population
Source: PLoS One. 2016 Oct 20;11(10):e0163877. doi: 10.1371/journal.pone.0163877 (PMC5072673; doi:10.1371/journal.pone.0163877)
Supplement: S2 Text — (DOCX) [file pone.0163877.s017.docx]

## Supporting information: S2 Text

## Supplementary References

References cited in Supporting information files are given below.

1. Nakajima, M., Takahashi, A., Kou, I., Rodriguez-Fontenla, C., Gomez-Reino, J.J., Furuichi, T., Dai, J., Sudo, A., Uchida, A., Fukui, N. *et al.* (2010) New sequence variants in HLA class II/III region associated with susceptibility to knee osteoarthritis identified by genome-wide association study. *PLoS One*, **5**, e9723.

2. Videman, T., Saarela, J., Kaprio, J., Nakki, A., Levalahti, E., Gill, K., Peltonen, L. and Battie, M.C. (2009) Associations of 25 structural, degradative, and inflammatory candidate genes with lumbar disc desiccation, bulging, and height narrowing. *Arthritis Rheum*, **60**, 470-481.

3. Noponen-Hietala, N., Kyllonen, E., Mannikko, M., Ilkko, E., Karppinen, J., Ott, J. and Ala-Kokko, L. (2003) Sequence variations in the collagen IX and XI genes are associated with degenerative lumbar spinal stenosis. *Ann Rheum Dis*, **62**, 1208-1214.

4. Raitakari, O.T., Juonala, M., Ronnemaa, T., Keltikangas-Jarvinen, L., Rasanen, L., Pietikainen, M., Hutri-Kahonen, N., Taittonen, L., Jokinen, E., Marniemi, J. *et al.* (2008) Cohort profile: the cardiovascular risk in Young Finns Study. *Int J Epidemiol*, **37**, 1220-1226.

5. Kaila-Kangas, L. (2007) *Musculoskeletal disorders and diseases in Finland. Results of the Health 2000 Survey*. National Public Health Institute, Helsinki, Finland.

6. Aromaa, A. and Koskinen, S. (2004) *Health and functional capacity in Finland: Baseline results of the Health 2000 Health examination survey*. National Public Health Institute, Helsinki, Finland.

7. Perttila, J., Merikanto, K., Naukkarinen, J., Surakka, I., Martin, N.W., Tanhuanpaa, K., Grimard, V., Taskinen, M.R., Thiele, C., Salomaa, V. *et al.* (2009) OSBPL10, a novel candidate gene for high triglyceride trait in dyslipidemic Finnish subjects, regulates cellular lipid metabolism. *J Mol Med (Berl)*, **87**, 825-835.

8. Vartiainen, E., Laatikainen, T., Peltonen, M., Juolevi, A., Mannisto, S., Sundvall, J., Jousilahti, P., Salomaa, V., Valsta, L. and Puska, P. (2010) Thirty-five-year trends in cardiovascular risk factors in Finland. *Int J Epidemiol*, **39**, 504-518.

## Web Resources

Cardiovascular Risk in Young Finns Study, <http://youngfinnsstudy.utu.fi/>

Health 2000 Study, <http://www.nationalbiobanks.fi/index.php/studies2/8-health2000>

FINRISK, <http://www.nationalbiobanks.fi/index.php/studies2/7-finrisk>

The 1000 Genomes Browser, <http://browser.1000genomes.org/index.html>

Finnish Care Register for Health Care, https://www.thl.fi/fi/web/thlfi-en/statistics/information-on-statistics/register-descriptions/care-register-for-health-care
